# The secRNome of Listeria monocytogenes Harbors Small Noncoding RNAs That Are Potent Inducers of Beta Interferon

**DOI:** 10.1128/mBio.01223-19

**Published:** 2019-10-08

**Authors:** Renate Frantz, Lisa Teubner, Tilman Schultze, Luigi La Pietra, Christin Müller, Konrad Gwozdzinski, Helena Pillich, Torsten Hain, Michaela Weber-Gerlach, Georgios-Dimitrios Panagiotidis, Ahmed Mostafa, Friedemann Weber, Manfred Rohde, Stephan Pleschka, Trinad Chakraborty, Mobarak Abu Mraheil

**Affiliations:** a Institute of Medical Microbiology, German Center for Infection Research (DZIF), Partner Site Giessen-Marburg-Langen, Justus-Liebig University Giessen, Giessen, Germany; b Institute of Medical Virology, Justus-Liebig University Giessen, Giessen, Germany; c Institute for Virology, FB10-Veterinary Medicine, Justus-Liebig University Giessen, Giessen, Germany; d Central Facility for Microscopy, Helmholtz Centre for Infection Research, Braunschweig, Germany; GSK Vaccines

**Keywords:** *Listeria monocytogenes*, type I IFN, secreted RNA

## Abstract

Cellular sensing of bacterial RNA is increasingly recognized as a determinant of host-pathogen interactions. The intracellular pathogen Listeria monocytogenes induces high levels of type I interferons (alpha/beta interferons [IFN-α/β]) to create a growth-permissive microenvironment during infection. We previously demonstrated that RNAs secreted by L. monocytogenes (comprising the secRNome) are potent inducers of IFN-β. We determined the composition and diversity of the members of the secRNome and found that they are uniquely enriched for noncoding small RNAs (sRNAs). Testing of individual sRNAs for their ability to induce IFN revealed several sRNAs with this property. We examined ril32, an intracellularly expressed sRNA that is highly conserved for the species L. monocytogenes and that was the most potent inducer of IFN-β expression of all the sRNAs tested in this study, in more detail. The rli32-induced IFN-β response is RIG-I (retinoic acid inducible gene I) dependent, and cells primed with rli32 inhibit influenza virus replication. We determined the rli32 motif required for IFN induction. rli32 overproduction promotes intracellular bacterial growth, and a mutant lacking rli32 is restricted for intracellular growth in macrophages. rli32-overproducing bacteria are resistant to H_2_O_2_ and exhibit both increased catalase activity and changes in the cell envelope. Comparative transcriptome sequencing (RNA-Seq) analysis indicated that ril32 regulates expression of the *lhrC* locus, previously shown to be involved in cell envelope stress. Inhibition of IFN-β signaling by ruxolitinib reduced rli32-dependent intracellular bacterial growth, indicating a link between induction of the interferon system and bacterial physiology. rli32 is, to the best of our knowledge, the first secreted individual bacterial sRNA known to trigger the induction of the type I IFN response.

## Abstract (precis)

Interferons are potent and broadly acting cytokines that stimulate cellular responses to nucleic acids of unusual structures or locations. While protective when induced following viral infections, the induction of interferons is detrimental to the host during L. monocytogenes infection. Here, we identify specific sRNAs, secreted by the bacterium, with the capacity to induce type I IFN. Further analysis of the most potent sRNA, rli32, links the ability to induce RIG-I-dependent induction of the type I IFN response to the intracellular growth properties of the bacterium. Our findings emphasize the significance of released RNA for *Listeria* infection and shed light on a compartmental strategy used by an intracellular pathogen to modulate host responses to its advantage.

## INTRODUCTION

Mammalian cells detect invading pathogens by recognizing “non-self” bacterial structures (pathogen-associated molecular patterns [PAMPs]) through pattern recognition receptors (PRRs). While recognition of many PAMPs such as peptidoglycan, lipopolysaccharide, and flagellin does not necessarily indicate an active infection, bacterial mRNA is a viability-associated PAMP (vita-PAMP) present only in viable bacteria (1).

Type I interferons (IFNs), which comprise several IFN-α subtypes, the single IFN-β subtype, and several other subtypes (IFN-ε, IFN-κ, IFN-ω, IFN-τ, IFN-ζ, and IFN-δ), are critical components of the antiviral defense of mammalian cells (2, 3). The induction of type I IFN can be achieved either by membrane-anchored Toll-like receptors which recognize microbial PAMPs in the extracellular or endosomal space (4) or through cytosolic PRRs such as RIG-I (retinoic acid inducible gene I)-like receptors which allow for immune surveillance in the cytoplasm (5). Unlike its role in virus defense, induction of the type I IFN response leads to an increase of host susceptibility to intracellular pathogens such as Listeria monocytogenes (6, 7), Mycobacterium tuberculosis (8, 9), and Francisella tularensis (10).

L. monocytogenes is a ubiquitously occurring facultative intracellular Gram-positive bacterium. It is the causative agent of listeriosis, a disease of low incidence but of considerable mortality (11). L. monocytogenes infects a variety of phagocytic and nonphagocytic host cells in which it can replicate intracellularly and spread from cell-to-cell (12). It is well known that, unlike heat‐killed L. monocytogenes or mutant bacteria that fail to enter the cytosol, viable *Listeria* that gain access to the cytosol generate protective CD8 T‐cell immunity (13, 14). A primary characteristic of this pathogen is its ability to deliver microbial molecules into the host cell cytosol. Once in the host cell, secreted effector molecules perform a variety of functions that contribute to pathogenesis.

Second messengers of bacteria, such as cyclic‐di‐AMP and cyclic‐di‐GMP, are actively secreted by replicating cytosolic *Listeria* and induce IFN-β production through the signaling molecule STING (stimulator of interferon genes) (15). This is also the case for secreted (but not cytosolic) L. monocytogenes RNA (comprising the secRNome), which triggers a strong IFN-β response (16). Secreted RNA was shown to be present in the host cytoplasm following infection with L. monocytogenes (17).

Type I IFN induction is detrimental to the host during *Listeria* infection. For example, mice lacking type I IFN receptors (*Ifnar^−/−^* mice) show greater resistance to *Listeria* infections than wild-type mice (6, 7). During the course of L. monocytogenes infection, high levels of type I IFN antagonize IFN-γ signaling by downregulating the interferon gamma receptor (IFNGR) on antigen-presenting cells (APCs) (18), increase lymphocyte apoptosis (6), enhance macrophage cell death (19), reduce the production of protective interleukin-12 (IL-12) and tumor necrosis factor alpha (TNF-α) (7), and inhibit neutrophil migration (20), thereby creating a microenvironment for bacterial growth. Thus, type I IFN-mediated modulation by L. monocytogenes is detrimental to the host and advantageous to the pathogen. Recently, a direct role for type I IFNs in bacterial survival has been suggested. Induction of type-1 IFNs promoted ActA polarization and actin-based motility of L. monocytogenes in the host cytosol and thereby enhanced cell-to-cell spread (21).

In this study, secreted RNAs of L. monocytogenes, present in two extracellular subcompartments, were identified. We have operationally divided the secRNome into the supernatant and the membrane vesicle (MV) fractions. The cargo of bacterial membrane vesicles (MVs), which are released during all phases of bacterial growth and comprise peptidoglycan, lipopolysaccharides, proteins, toxins, and nucleic acids, was previously shown to exploit different mechanisms to enter the host cells (22, 23). Recently, it was demonstrated that L. monocytogenes produces MVs both *in vitro* and *in vivo* (24).

We found that specific RNAs, in particular, noncoding small RNAs (sRNAs), are enriched in the secreted fraction. Screening of individual sRNAs led to detection and identification of several sRNAs that specifically induce type I IFN. In-depth characterization of one of these sRNAs, viz., rli32, revealed features for RIG-I (retinoic acid inducible gene I) activation and for the functional activation of the immune response in tissue culture cell lines as well as in a mouse model. Furthermore, overexpression and deletion of rli32 affected both IFN-β production and intracellular bacterial growth.

## RESULTS

### Discovery of L. monocytogenes sRNA rli32 as a potent IFN-β inducer.

**FIG 1:**
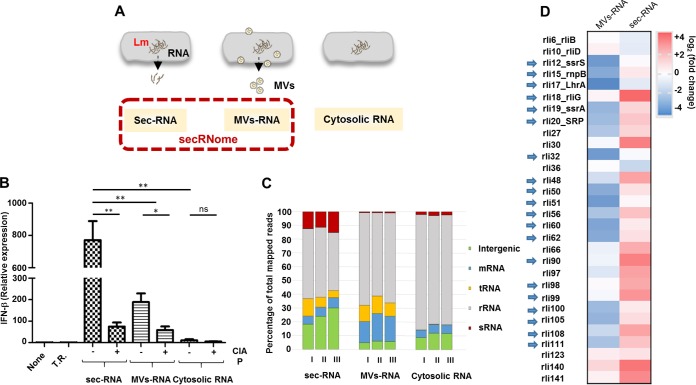
secRNome of L. monocytogenes. (A) The secRNome of L. monocytogenes comprises two subcompartments: “naked” RNA (sec-RNA) and RNA associated with membrane vesicles (MVs), shed in the medium by bacteria. Cytosolic RNA pertains to the RNA inside the bacterial cell. Lm, wild-type strain. (B) IFN-β induction by sec-RNA and MV-RNA. Data represent levels of induction of IFN-β expression upon transfection of sec-RNA, MV-RNA, and cytosolic RNA in BMDM (100 ng/10^6^ cells). Transfection of sec-RNA and MV-RNA highly induced IFN-β expression (70-fold and 20-fold, respectively) compared to cytosolic RNA. Treatment of the three RNA fractions with CIAP (calf intestinal alkaline phosphatase) significantly reduced IFN-β expression levels in sec-RNA and MV-RNA. Cells treated with a transfection reagent (Lipofectamine) only were used as the control. Data are presented as means ± standard deviations (SD) of results from three experiments (ns, nonsignificant; *, *P < *0.05; **, *P < *0.01). T.R., transfection reagent only; None, untransfected. (C) The transcriptome of the secRNome. Data represent percentages of total reads of cytosolic RNA, MV-RNA, and sec-RNA mapped to the L. monocytogenes genome. Data represent results from three independent experiments (experiments I, II, and III). (D) Heat map of 30 sRNAs differentially expressed in sec-RNA and MV-RNA compared to cytosolic RNA of L. monocytogenes. The 20 sRNA candidates selected for *in vitro* transcription are indicated with arrows. Data represent means of results from three independent experiments.

We sought to define the composition of the secreted RNA fraction that was shown to be a strong inducer of IFN-β response (16). The secRNome of L. monocytogenes supernatant fluids is composed of “naked” RNA (sec-RNA) and intravesicular RNAs that are shed to the external medium by membrane vesicles (MV-RNA) (schematically depicted in Fig. 1A). sec-RNA and MV-RNA were isolated from exponentially grown cultures in defined minimal medium (see Fig. S1 in Data Set S1 in the supplemental material). To investigate potential cell lysis as a source of sec-RNA, the enzyme activity of strictly cytoplasmic aminopeptidase C (PepC) was assayed as previously described (25, 26). The results demonstrated an absence of PepC activity in the supernatant (Fig. S2 [Data Set S1]).

10.1128/mBio.01223-19.4 DATA SET S1 Fig. S1 to S12 and supplemental materials and methods. Download Data Set S1, DOCX file, 2.0 MB. Copyright © 2019 Frantz et al. 2019 Frantz et al. This content is distributed under the terms of the Creative Commons Attribution 4.0 International license.

Interestingly, the RNA profiles of L. monocytogenes sec-RNA and MV-RNA were significantly different from that of cytosolic RNA. Secreted fractions indicated an accumulation of short RNAs (i.e., RNAs less than 200 nucleotides [nt] in length). This was particularly true for the MV-RNA fraction (Fig. S3 [Data Set S1]).

Transfection of the three RNA fractions into bone marrow-derived macrophages (BMDM) demonstrated that sec-RNA and MV-RNA induced higher IFN-β levels than cytosolic RNA (70-fold and 20-fold, respectively) (Fig. 1B). Treatment of sec-RNA and MV-RNA fractions with CIAP (calf intestinal alkaline phosphatase), which catalyzes the hydrolysis of 5′ phosphate groups from RNA, led to a significant reduction in IFN-β expression following transfection in BMDM (Fig. 1B). This suggests the presence of a high proportion of 5′ triphosphate RNA in both fractions. Activation of the RNA receptor RIG-I by cytosolic-pathogen-derived RNA requires double-stranded blunt-end 5′ triphosphate RNA (27). In addition, RIG-I senses RNAs with complementary 5′ and 3′ ends that hybridize to form a hairpin, as found for many viral genomes (28, 29).

To identify the RNA species present in the sec-RNA and MV-RNA fractions, we performed transcriptome sequencing (RNA-Seq) on sec-RNA, MV-RNA, and cytosolic RNA. Panel C of Fig. 1 depicts the percentages of different RNA types detected among the total reads mapped to the L. monocytogenes genome. The sequencing data showed that sec-RNA and MV-RNA comprised all forms of RNA, including tRNA, rRNA, mRNA, and sRNA. Interestingly, the sequencing data revealed a high proportion of noncoding small RNAs (sRNA) in sec-RNA (12.9%) compared to cytosolic RNA (2.4%) and MV-RNA (0.7%). Furthermore, some individual sRNAs demonstrated even higher enrichment levels in sec-RNA and MV-RNA than in cytosolic RNA (Fig. 1D; see also Fig. S4 [Data Set S1]).

The relatively high abundance of sRNA reads in sec-RNA compared to cytosolic RNA prompted us to investigate the contribution of sRNA to the IFN-β induction by sec-RNA. We selected 20 sRNA candidates (highlighted in Fig. 1D) representing enriched sRNAs in sec-RNA such as rliG, rli48, rli90, rli99, and rli108 (Fig. 1D; see also Table S1 in the supplemental material). We also considered those sRNAs whose expression levels either were induced during intracellular growth (rli32, rli48, rli51, rli60, rli98, rli100, rli105, and rliG) or exhibited increased transcription during extracellular growth (rli50, rli99, rli108, rli111, LhrA, rnpB, SRP, ssrA, and ssrS) (30). In addition to sRNAs, two tRNAs, highly enriched in sec-RNA (lmot11-tRNA-Ser) and in MV-RNA (lmot67-tRNA-Val), were also studied (Table S1).

10.1128/mBio.01223-19.1 TABLE S1 RNA-Seq data of sec-RNA, MV-RNA, and cytosolic RNA. Download Table S1, XLSX file, 0.3 MB. Copyright © 2019 Frantz et al. 2019 Frantz et al. This content is distributed under the terms of the Creative Commons Attribution 4.0 International license.

**FIG 2:**
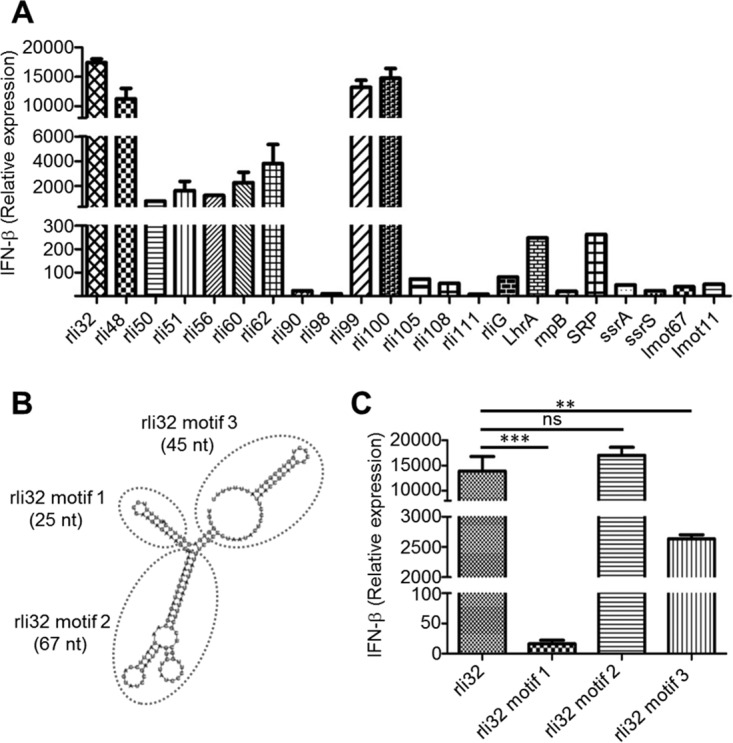
Individual transfection of the IVT sRNAs in BMDM cells revealed rli32, rli48, rli99, and rli100 as potent inducers of IFN-β. (A) IFN-β induction in BMDM cells by sRNAs detected in sec-RNA and MV-RNA. BMDM cells were transfected with different IVT sRNA molecules (50 ng/10^6^ cells). The IFN-β induction was assayed 6 h posttransfection by qRT-PCR. Data are presented as means ± SD of results from three experiments. Cells treated with the transfection reagent (Lipofectamine) only were used as the control. The results identified candidate motifs in rli32 essential for IFN-β induction. (B) Folding prediction of three candidate motifs of rli32 by the use of RNAfold ViennaRNA software. (C) The three motifs were transcribed *in vitro* and individually transfected in BMDM. Motif 2 showed the highest level of IFN-β induction, that level is similar to the level seen with the complete rli32 sequence. Data are presented as means ± SD of results from three experiments (ns, nonsignificant; **, *P* < 0.01; ***, *P* < 0.001).

The sRNAs were subjected to *in vitro* transcription (IVT) and individually transfected (50 ng) in BMDM and HEK293 cells. The results demonstrated that rli32, rli99, and rli100 triggered the strongest IFN-β response in BMDM and HEK293 cells whereas rli48, whose activity may be cell type dependent, showed high levels of IFN-β induction only in BMDM (Fig. 2A; see also Fig. S5A [Data Set S1]). Additional sRNAs such as LhrA, rli50, rli51, rli56, rli60, rli62, and SRP showed moderate IFN-β induction. The remaining sRNAs (rli90, rli98, rli105, rli108, rli111, rliG, rnpB, ssrA, and ssrS) revealed very weak IFN-β induction properties (Fig. 2A; see also Fig. S5A [Data Set S1]). We validated these findings using HEK-Blue IFN-α/β cells, which allow the detection of type I IFN protein levels by enabling monitoring of the activation of the interferon-stimulated gene factor 3 (ISGF3) pathway for evaluation of the correlation between mRNA and protein levels of IFN-β. The transfection of rli32 and ssrS (a very weak IFN inducer) in these reporter cells indicated similar IFN-β induction properties (Fig. S5B [Data Set S1]).

These results revealed that the ability to induce high IFN-β expression by a given RNA does not simply correlate with its abundance in the sec-RNA fraction, as found for, e.g., rliG, rli30, rli90, and rli108. These sRNAs induced only low levels of IFN-β expression after transfection in macrophages and HEK293 cells (Fig. 2A; see also Fig. S5A [Data Set S1]). We selected rli32 for further studies as it is intracellularly expressed (30) and highly specific for the species L. monocytogenes (Fig. S6 [Data Set S1]) and is the most potent sRNA species required for type I IFN induction (Fig. 2A; see also Fig. S5A [Data Set S1]). Moreover, it has recently been shown that expression of rli32 strongly depends on the transcriptional regulator VirR, a secondary regulator of virulence of L. monocytogenes (31).

### Characterization of essential rli32 motif for IFN-β induction.

We sought to identify those specific sequences/motifs of rli32 responsible for its strong immunostimulatory activity. We modeled the secondary structures of rli32 *in silico* using RNAfold WebServer (32). Three candidate motifs that were predicted for rli32 (Fig. 2B) were separately transcribed *in vitro* and transfected to determine their relative efficacies for IFN-β induction. Motif 2 induced the highest IFN-β production, which attained a level that was similar to that seen with the full-length rli32 sequence (Fig. 2C). Motif 3 induced lower levels than either rli32 or motif 2, whereas motif 1 failed to induce IFN-β. Structural modifications of motif 2 introduced by PCR-mediated *in vitro* sequence alteration mutagenesis led to a significant decrease in their ability to induce IFN-β response following transfection in BMDM (Fig. S7A and B [Data Set S1]).

### rli32-triggered IFN-β induction is RIG-I dependent.

**FIG 3:**
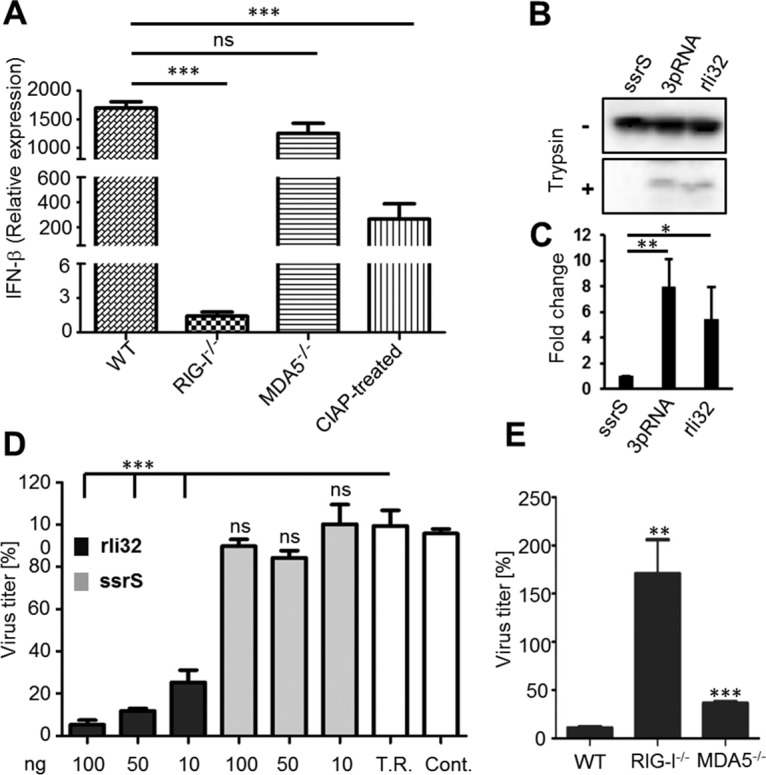
rli32-triggered IFN-β induction is RIG-I dependent. (A) Contribution of cytosolic receptors RIG-I and MDA5 to the recognition of rli32. Transfection of RIG-I^−/−^ and MDA5^−/−^ deficient HEK293 cells with rli32 revealed that the triggering of IFN-β expression by rli32 is RIG-I dependent. The absence of RIG-I abolished the expression of IFN-β almost completely. Treatment of rli32 with CIAP led to significantly reduced IFN-β expression after transfection in HEK293 cells. Cells treated with the transfection reagent (Lipofectamine) only were used as the control. WT, wild type. (B) Conformational switch of RIG-I. A549 cells transfected with ssrS, rli32, and 3pRNA, as a positive control, were lysed and treated with trypsin. Trypsin digestion of lysates from rli32-transfected and 3pRNA-transfected A549 cells resulted in the emergence of a protease-resistant RIG-I fragment, whereas the ssrS transfection resulted in rapid degradation of RIG-I. (C) Quantification of the differences in the band intensities of RIG-I following trypsin digestion depicted as fold changes. (D) Dose-dependent inhibition of influenza A virus replication by rli32. HEK293 cells were transfected with three different concentrations of rli32 and ssrS (10, 50, and 100 ng) 24 h prior to infection with A/PR/8/34 (H1N1).Transfection of 100 ng rli32 diminished the virus titer by as much as 95%, while transfection of only 10 ng resulted in a reduction of 75%. On the other hand, the pretreatment with the weak IFN-β inducer ssrS did not inhibit virus proliferation. T.R., transfection reagent; Cont., untreated control). (E) Contribution of the cytosolic receptors RIG-I and MDA5 to the rli32-triggered antiviral response. rli32 was transfected into RIG-I^−/−^ and MDA5^−/−^ deficient HEK293 cells, and the respective supernatants were used for virus titer assay. The virus titer was unaffected in HEK293 cells deficient for RIG-I, whereas the pretreatment of MDA5^−/−^ deficient HEK293 cells with rli32 led to a strong decrease of the virus titer. Data are presented as means ± SD of results from three experiments (ns, nonsignificant; *, *P < *0.05; **, *P < *0.01; ***, *P < *0.001).

As cellular PRRs RIG-I and the related MDA5 (melanoma differentiation-associated gene 5) are involved in sensing of *Listeria* RNA (16), we investigated the contribution of these sensors in recognition of rli32. For this purpose, we used RIG-I^−/−^ and MDA5^−/−^ deficient HEK293 cells. Transfection of IVT rli32 in RIG-I^−/−^ and MDA5^−/−^ deficient HEK293 cells revealed that the IFN-β expression triggered by rli32 depends mainly on the presence of RIG-I. Transfection of rli32 in RIG-I^−/−^ deficient HEK293 cells abolished the expression of IFN-β almost completely (Fig. 3A). However, the absence of MDA5 did not lead to a significant reduction in IFN-β expression in HEK293 cells (Fig. 3A). Treatment of rli32 with CIAP strongly reduced IFN-β expression after transfection in HEK293 cells, indicating that rli32 is a RIG-I ligand.

### Activation of RIG-I signaling by rli32.

Following binding of ligand to RIG-I, a conformational change is induced that renders the protein resistant to limited trypsin digestion (33–36). We examined our data for changes in RIG-I conformation following transfection of A549 cells with IVT rli32 RNA. The A549 human cell line has been established for monitoring activation of RIG-I (33). Another sRNA, ssrS, was selected as a negative control due to its very low capacity to induce IFN-β response (Fig. 2A; see also Fig. S5A [Data Set S1]). A short double-strand RNA bearing 5′ triphosphate, namely, 3pRNA, a validated RIG-I ligand, was used as a positive control. The results showed that trypsin digestion of lysates from ssrS-transfected A549 cells indicated degradation of RIG-I, whereas transfection with either rli32 or 3pRNA produced the protease-resistant RIG-I fragment indicative of ligand binding (Fig. 3B). Quantification of the differences in band intensities of RIG-I following trypsin digestion is depicted in Fig. 3C.

### Inhibition of influenza A virus propagation by rli32.

As RIG-I is an essential receptor for sensing many viruses, including influenza virus (37, 38), we next explored the capacity of IFN-β induced by rli32 in HEK293 cells to inhibit the propagation of influenza virus [A/PR/8/34 (H1N1, PR8)]. Supernatants from HEK293 cells pretreated with different rli32 concentrations (10, 50, and 100 ng) and subsequently infected with the virus were used to determine their effect on influenza virus propagation in MDCK-II cells. The same was done using ssrS as a control. Pretreatment with rli32 strongly reduced the virus titer in a concentration-dependent manner (Fig. 3D). Transfection of 100 ng rli32 diminished the virus titer by up to 95%, with a 75% reduction already obtained at 10 ng (Fig. 3D). On the other hand, supernatants from cells pretreated with the weak IFN-β inducer ssrS did not inhibit virus proliferation. These results demonstrate the functionality of IFN-β induced by rli32.

To investigate the contribution of the cytosolic receptors RIG-I and MDA5 to the rli32-triggered antiviral response, rli32 was transfected into RIG-I^−/−^ and MDA5^−/−^ deficient HEK293 cells and the respective supernatants were used for virus titer assay. The virus titer was unaffected in HEK293 cells deficient for RIG-I, whereas pretreatment of MDA5^−/−^ deficient HEK293 cells using rli32 led to a strong decrease in virus titer (Fig. 3E). This finding confirms the results observed with IVT rli32 transfected in RIG-I^−/−^ HEK293 cells, where an IFN-β response was not observed (Fig. 3A).

### Increased IFN-β levels of recombinant bacteria overproducing rli32.

**FIG 4:**
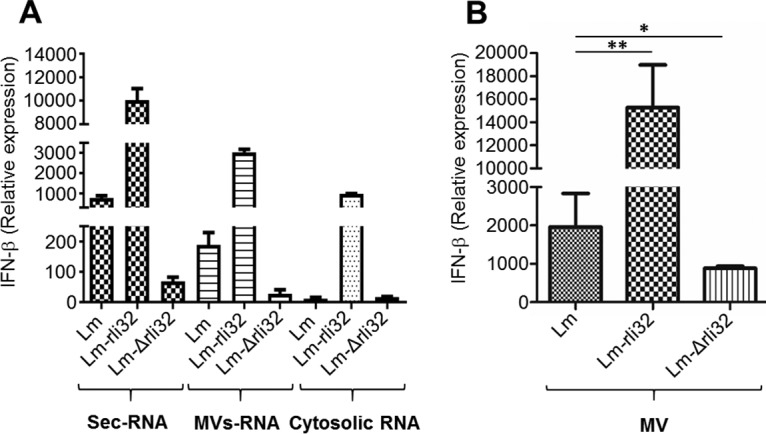
Impact of rli32 deletion and overproduction on IFN-β induction. (A) sec-RNA, MV-RNA, and cytosolic RNA were isolated from wild-type L. monocytogenes, strain Lm-rli32 (L. monocytogenes rli32-overproducing strain), and strain Lm-Δrli32 (L. monocytogenes Δrli32 deletion mutant). Transfection of the three RNA fractions (sec-RNA, MV-RNA, and cytosolic RNA) isolated from Lm-rli32 induced a higher IFN-β response in macrophages than those from L. monocytogenes and Lm-Δrli32. SecRNA and MV-RNA isolated from the strain lacking rli32 showed a significant decrease in the IFN-β response. Cells treated with the transfection reagent (Lipofectamine) only were used as the control. (B) Addition of membrane vesicles (MVs) isolated from the supernatant of the Lm-rli32 strain to macrophages showed that those MVs were more potent in inducing an IFN-β response than the MVs from the wild-type strain or strain Lm-Δrli32. Data are presented as means ± SD of results from three experiments (*, *P < *0.05; **, *P < *0.01; ***, *P < *0.001).

We next evaluated the role of bacterially expressed rli32 in induction of an IFN-β response. To address the consequences of both the lack of rli32 and overexpression of rli32 in the sec-RNA, MV-RNA, and cytosolic RNA fractions with respect to induction of an IFN-β response, all three RNA fractions were isolated from wild-type L. monocytogenes, strain Lm-rli32 (L. monocytogenes rli32-overproducing strain), and strain Lm-Δrli32 (L. monocytogenes Δrli32 deletion mutant). Neither overexpression nor deletion of rli32 led to overall changes in the total amount of secreted RNA. Transfection of sec-RNA, MV-RNA, and cytosolic RNA isolated from the Lm-rli32 strain induced a strong IFN-β response in macrophages (Fig. 4A).

We assessed the amount of rli32 transcripts in the three RNA fractions isolated from the respective overproducing strains using reverse transcription-quantitative PCR (qRT-PCR). The results showed higher numbers of transcripts of rli32 in all three RNA fractions than in the wild type (Fig. S8A [Data Set S1]). This demonstrates that bacterially expressed rli32 is, like IVT-generated rli32 (Fig. 2A; see also Fig. S5A [Data Set S1]), a potent inducer of IFN-β.

In contrast to the results seen with rli32 overexpression, the absence of rli32 strongly reduced the capacity of the sec-RNA, MV-RNA, and cytosolic RNA fractions to induce IFN-β (Fig. 4A). However, production of rli32 under the control of its native promoter in the deletion mutant increased the IFN-β induction capabilities of sec-RNA isolated from the complemented mutant to a level comparable to that seen with the wild type (Fig. S8B [Data Set S1]).

MVs isolated from the supernatant of the rli32-overproducing strain added to macrophages were highly active in inducing IFN-β response and were more active than MVs isolated from either the wild type or the corresponding isogenic Δrli32 deletion mutant (Fig. 4B).

### Rli32 promotes intracellular survival of L. monocytogenes.

**FIG 5:**
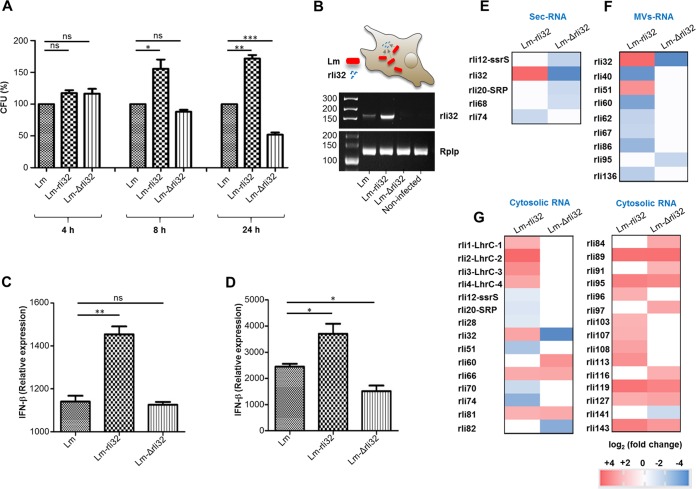
Rli32 affects the intracellular growth of L. monocytogenes. Macrophages were infected with the parental strain (Lm), rli32-overproducing strain (Lm-rli32), and Δrli32 deletion mutant (Lm-Δrli32). (A) The number of intracellularly grown bacteria (quantified as CFUs) was counted on agar plates following lysis of the macrophages at 4, 8, and 24 h p.i. The data from time points 8 and 24 h p.i. revealed increased growth of Lm-rli32 over that of the wild type. The level of growth of Lm-Δrli32 was reduced compared to that of the parental strain at 24 h p.i. Data are presented as means ± SD of results from three experiments. (B) Amounts of rli32 transcripts in the cytosol of macrophages infected with the Lm, Lm-rli32, and Lm-Δrli32 strains. The cytosolic host RNA was isolated 8 h p.i. and reverse transcribed in cDNA, followed by amplification using qRT-PCR. The bands were separated on an agarose gel. As the control, the amount of the host reference gene Rplp in the cytosolic host RNA was determined. (C and D) Induction and repression of IFN-β response during infection with strains Lm-rli32 and Lm-Δrli32. Expression of IFN-β in macrophages infected with strains Lm, Lm-rli32, and Lm-Δrli32 was determined at 8 h p.i. (C) and 24 h p.i. (D). Data are presented as means ± SD of results from three experiments (ns, nonsignificant; *, *P < *0.05; **, *P < *0.01; ***, *P < *0.001). (E to G) Heat map representing ratios of differences in the compositions of sRNAs in (E) sec-RNA, (F) MV-RNA, and (G) cytosolic RNA in strains Lm-rli32 and Lm-Δrli32 in comparison to strain Lm.

To assess a role of rli32 in intracellular growth of L. monocytogenes, the ability of the Lm-Δrli32 and Lm-rli32 strains to grow intracellularly in BMDM macrophages was examined at 4, 8, and 24 h postinfection (p.i.). Intracellular growth of the Lm-rli32 strain was already enhanced at 8 h p.i., and significant restriction of intracellular growth was detected for the deletion mutant at 24 h p.i. (Fig. 5A). Neither the deleted strain nor the overproducing strain exhibited differences under conditions of growth *in vitro* (Fig. S9A [Data Set S1]). The increased amounts of rli32 transcripts detected in the cytosol of macrophages infected with Lm-rli32 (Fig. 5B) correlated with an increase of the IFN-β response seen at 8 h p.i. (Fig. 5C). Similarly, the reduction in the level of IFN-β expression observed with the Lm-Δrli32 strain was associated with its poor growth at 24 h p.i. (Fig. 5D). The restricted intracellular growth of the deletion mutant was restored to the level of the wild type through complementation with rli32 expressed under the control of the rli32 native promoter (Fig. S9B [Data Set S1]).

### Physiological consequences of rli32 for the growth of L. monocytogenes.

We used transcriptome-based analyses to address the physiological consequences of rli32 overexpression or deletion for the growth of L. monocytogenes and to systematically elucidate changes in the RNA composition in sec-RNA, MV-RNA, and cytosolic RNA in the wild-type (Lm), Lm-rli32, and Lm-Δrli32 strains. Remarkably, Lm-rli32 and Lm-Δrli32 showed only minor differences in the sRNA composition of the sec-RNA and MV-RNA fractions compared to wild-type L. monocytogenes at a fold change (fc) cutoff value of ≥2. The only enriched sRNA species in the sec-RNA fraction of Lm-rli32 was rli32 (+5.5 fc) (Fig. 5E). sRNAs ssrS, SRP, and rli68 were downregulated in the deletion mutant (Fig. 5E). Transfection of IVT ssrS and SRP showed that they are weak IFN-β inducers (Fig. 2A; see also Fig. S5A [Data Set S1]). Rli68 (−2.17 fc) has not yet been investigated. Similar results were observed in the MV-RNA fractions. As expected, rli32 levels were elevated (+4.90 fc) in strain Lm-rli32 (Fig. 5F). The amount of rli51 was also increased (+3.32 fc). This sRNA was among the individually tested *in vitro* transcribed candidates that indicated moderate induction of IFN-β (Fig. 2A; see also Fig. S5A [Data Set S1]). Several other rli sRNAs (rli40, rli51, rli60, rli62, rli67, rli86, and rli136) were found to be downregulated (Fig. 5F). In the Lm-Δrli32 mutant, only rli95 was downregulated (−2.25 fc) (Fig. 5F).

Major differences were detected in the cytosolic mRNA fraction (Fig. S10A to C [Data Set S1]). For example, the data show induction of several genes (lmo1958, lmo1960m, and lmo2181 to lmo2186) encoding a ferrichrome ABC transporter in strain Lm-rli32 (Fig. S10C [Data Set S1]; see also Table S2). Also noteworthy is the downregulation of the complete tryptophan operon (lmo1627 to 1633) in the Lm-Δrli32 mutant (Fig. S10C [Data Set S1]). Further work will be required to elucidate the importance of these changes.

10.1128/mBio.01223-19.2 TABLE S2 Differentially expressed mRNAs in sec-RNA, MV-RNA, and cytosolic RNA in the overexpression strain (Lm-rli32) and deletion mutant (Lm-Δrli32) compared to the wild type (Lm). Download Table S2, PDF file, 0.3 MB. Copyright © 2019 Frantz et al. 2019 Frantz et al. This content is distributed under the terms of the Creative Commons Attribution 4.0 International license.

We found that 17 sRNAs were upregulated and 6 sRNAs were significantly downregulated (Fig. 5G). In the Lm-Δrli32 deletion mutant, expression of 12 sRNAs was induced and 3 sRNAs were downregulated (Fig. 5G). Additionally, seven sRNAs (rli66, rli81, rli89, rli95, rli119, rli127, and rli143) were found to be upregulated in the cytosol of both the Lm-rli32 and Lm-Δrli32 strains (Fig. 5G).

**FIG 6:**
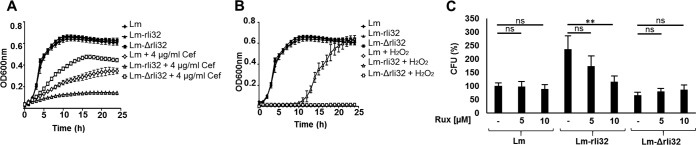
Physiological consequences of rli32 for the growth of L. monocytogenes. Bacterial growth in the presence of cefuroxime and H_2_O_2_ was assayed. (A and B) Impact of (A) cefuroxime (Cef) (4 μg/ml) and (B) H_2_O_2_ (0.15%) on the growth of strains Lm, Lm-Δrli32, and Lm-rli32. (C) Impact of the IFN signaling inhibitor ruxolitinib (Rux) (1 μM and 10 μM) on the growth (24 h p.i.) of strains Lm, Lm-rli32, and Lm-Δrli32 in macrophages. Data are presented as means ± SD of results from three experiments (ns, nonsignificant; **, *P < *0.01).

We detected elevated expression of sRNAs LhrC1, LhrC2, LhrC3, and LhrC4 (LhrC1–4) in the cytosolic fraction of Lm-rli32 (Fig. 5G). As sRNAs LhrC1–4 have previously been implicated in changes in membrane composition, we examined the association of rli32 and cell envelope stress. Previous studies have shown that the beta-lactam antibiotic cefuroxime (a cephalosporin), which strongly affects the integrity of the L. monocytogenes cell envelope, induces expression of the sRNAs LhrC1–5 (39–41). The recombinant overexpressing rli32 was more sensitive than the parental strain in the presence of cefuroxime (4 μg/ml), whereas the Lm-Δrli32 deletion mutant strain was more resistant to cefuroxime and grew better than the parental strain (Fig. 6A).

### Rli32 overexpression enhances resistance to H_2_O_2_.

As reactive oxygen species (ROS) are key components of the macrophage response to invading pathogens, we tested the impact of different oxidative stress-inducing agents such as hydrogen peroxide (H_2_O_2_), paraquat (PQ), and ethanol on the growth of the Lm-rli32 and Lm-Δrli32 strains. Additionally, we examined the impact of low pH values (pH 2.5 and 5) on the growth of the three strains. We found that Lm-rli32 was more growth resistant to a high (0.15%) H_2_O_2_ concentration than both Lm-Δrli32 and the parental strain, whose growth was completely inhibited (Fig. 6B). In addition, there was an increase in catalase production in Lm-rli32 compared to both Lm-Δrli32 and the parental strain (Fig. S11B [Data Set S1]). There were no differences in growth properties seen either with the other tested oxidative stress-inducing agents tested or with conditions simulating low pH (Fig. S11A to F [Data Set S1]). Thus, the increased resistance to H_2_O_2_ in the rli32-overproducing strain contributes to intracellular survival of Lm-rli32.

### Rli32 promoted intracellular survival requires type I IFN.

To examine the relation between the rli32-induced type I IFN response and intracellular growth of L. monocytogenes, we treated macrophages with ruxolitinib (1 μM and 10 μM), an inhibitor of IFN-signaling (42), using the infection conditions described for the experiment represented in Fig. 5A. Addition of increasing concentrations of inhibitor (1 and 10 μM) reduced the intracellular growth of the rli32-overproducing recombinant Lm-rli32 strain to a level similar to that of the wild-type strain (Fig. 6C). No significant changes in the levels of growth of the parental and Lm-Δrli32 strains were observed (Fig. 6C). Since RIG-I is the main cytosolic receptor of rli32 (Fig. 3A), we also examined the growth properties of Lm-rli32 and Lm-Δrli32 in RIG-I^−/−^ deficient HEK293 cells. No significant changes in the intracellular growth characteristics of all of the strains examined were observed in RIG-I^−/−^ deficient HEK293 cells (Fig. S12A [Data Set S1]). In the wild-type HEK293 cells, growth of the Lm-rli32 strain was enhanced, as expected, whereas that of the Lm-Δrli32 strain was reduced (Fig. S12B [Data Set S1]). These results demonstrate that rli32-dependent induction of the IFN-β level is coupled to intracellular growth of L. monocytogenes.

## DISCUSSION

Sensing of pathogen-derived nucleic acids from bacteria growing in the cytosol of infected host cells has emerged as a major mechanism for innate immune activation during infection. It was previously reported that an augmented type I IFN response and NLRP3 inflammasome-dependent activation are triggered by bacterial RNA for Gram-negative bacteria (1). However, there is currently little detailed information on the nature and the types of RNAs involved in the modulation of the immune response to bacterial infections. RNAs represent surrogate molecules of bacterial viability and are sensed as vita-PAMPs by the host innate immune system.

Secreted extracellular RNA is present in at least two subcellular fractions: the bacterial supernatant and MVs released by bacteria. As MVs are able to deliver virulence factors, including toxins and immunomodulatory molecules, directly into the host cells during infection, they represent a new dimension for host-pathogen interactions (24, 43). Outer membrane vesicles (OMVs) released by Pseudomonas aeruginosa and other bacteria such as Porphyromonas gingivalis and Treponema denticola also contain such sRNAs that have immune suppressive activities (44, 45). In contrast, because of controversy regarding the origins of secreted RNA, much less is known about the role of RNA present in this compartment.

We used RNA-Seq to provide insight into the composition and diversity of RNA species and to perform comparisons to whole-cell-derived RNA. Characterization of the secRNome in L. monocytogenes showed that, apart from the presence of structural noncoding RNAs (ribosomal RNAs), there was unique enrichment of noncoding nonstructural sRNAs. Three of the sRNAs detected in the secRNAome of L. monocytogenes (rli50, rli60, and LhrA) have been previously characterized. Isogenic mutants that lack rli50 or rli60 are significantly attenuated for intracellular survival and proliferation in macrophages (30, 46). LhrA is an Hfq-dependent posttranscriptional regulator affecting the expression of nearly 300 genes in L. monocytogenes (47).

Following the identification of sRNAs present in sec-RNA and MV-RNA fractions, a systematic screening approach was used to identify those molecules that are potent inducers of type I IFN response. This led to the detection of rli32, rli99, and rli100 as the most potent inducers of type I IFN. We targeted our efforts at the characterization of rli32, as it is expressed intracellularly (30), was the most potent inducer of IFN-β responses among the sRNAs tested, and is highly conserved and unique to L. monocytogenes.

Recent findings indicate that transcription of rli32 is part of the repertoire of genes controlled by VirR, a transcriptional regulator of virulence genes in L. monocytogenes (31). rli32 is also part of AgrA regulon, a communication system involved in adaptation of L. monocytogenes to its environment and during infection (48–50). Expression of rli32 was shown previously to be significantly reduced in a mutant strain lacking the response regulator AgrA (51). Finally, rli32 was also found previously to be one of a number of sRNAs that interact with the RNA binding protein SpoVG (52). Nevertheless, a role for rli32 in bacterial physiology has not been directly addressed.

Deletion of rli32 impairs intracellular growth of L. monocytogenes in macrophages, while its overproduction promotes intracytosolic replication. We found that there were graded responses among the strains tested for resistance to H_2_O_2_, with the rli32-overproducing strain showing the highest level of resistance. As we show here, a major component of this resistance was due to increased catalase activity. Production of cytosolic H_2_O_2_ during L. monocytogenes infection is required for proinflammatory signaling leading to cytokine secretion (53).

Expression of rli32 has also affects the composition of the bacterial cell wall. Strains producing rli32 exhibit enhanced sensitivity to the cephalosporin cefuroxime, an effect that is probably associated with its ability to regulate the production of the sRNAs LhrC1–5. Previous studies have shown that cefuroxime affects the integrity of L. monocytogenes cell envelope and induces the expression of the sRNAs LhrC1–5 (39). The elevated expression levels of the four known sRNAs LhrC1, LhrC2, LhrC3, and LhrC4 (LhrC1–4) contribute to the enhanced intracellular growth of L. monocytogenes. Multicopy sRNAs LhrC1–4 are known to be highly induced during growth in blood (54), during intracellular growth (30), and in response to both heme toxicity and envelope stress (39–41).

Current evidence suggests that induction of type I IFN is closely linked to physiological properties of intracellular L. monocytogenes. Thus, induction of type I IFN promotes polarization of its actin assembly-inducing ActA protein on the bacterial surface to enhance intracytosolic motility and promote cell-to-cell spread (21). Here, we show that inhibiting IFN signaling restricts intracellular growth of L. monocytogenes even when rli32 is overexpressed. This suggests that the cues resulting from modulation of cellular pathways following rli32-dependent IFN-β induction that are required for changes in the bacterial envelope to facilitate bacterial growth are no longer sensed. Further studies are required to identify the IFN-dependent host cell processes involved and the nature of the cues sensed by intracellular bacteria.

The presence of multiple secreted vita-PAMPS, such as either cyclic-di-AMP (c-di-AMP) or any of the various sRNAs described here, that target multiple cellular receptors, viz., STING and RIG-I, for inducing a type I interferon response implies an intrinsic need for live bacteria to create an environment that allows sufficient growth as a beachhead for further dissemination during infection. Further work will be required to address the role(s) of the other sRNAs detected. At least one species, rli48, exhibits cell-type specificity in the induction of the IFN response, and additional studies are required to address whether common and overlapping sRNAs are used, depending on type of cell infected. The diversity of sRNAs in a single bacterium inducing type I interferon signaling is intriguing. Thus, while c-di-AMP may be seen as a common vita-PAMP produced by many bacteria, the various sRNAs described here could be classified as variable vita-PAMPs that provide diversity that shapes both bacterial and cell type specificity.

In summary, we provide direct evidence that secreted individual bacterial sRNAs have the capacity to induce type I IFN. However, the mechanisms for RNA secretion and sorting in the supernatants and MVs remain poorly understood and require further study. We describe rli32, which, to the best of our knowledge, is the first secreted individual bacterial sRNA that links RIG-I-dependent induction of the type I IFN response to intracellular growth of the bacterium. The diversity of RNAs deriving from a single bacterial species that target RIG-I-dependent type I interferon induction was unexpected, and the approach employed here can be used to identify vita-RNA species in other bacteria that contribute to immune sensing and evasion during infection.

## MATERIALS AND METHODS

### Bacterial strains and growth conditions.

L. monocytogenes strain EGD-e (GenBank accession no. NC_003210) (55) was used in this study. Bacteria were grown in BHI (brain heart infusion) broth (VWR) overnight at 37°C with shaking at 180 rpm (Unitron incubator; Infors). L. monocytogenes overnight cultures were diluted 1:50 in 2 liters of fresh defined minimal medium broth (MM) (56) and incubated at 37°C until the exponential growth phase (optical density at 600 nm [OD_600_] = 1.0). Escherichia coli DH10β cells (Invitrogen) were grown in Luria-Bertani (LB) broth containing 300 μg/ml erythromycin to select for the plasmid pERL3 (57). L. monocytogenes strains harboring pERL3 were grown in the presence of 5 μg/ml erythromycin. Primers used for the construction of rli32 overexpression vector and the rli32 deletion mutant are listed in Table S3 in the supplemental material.

10.1128/mBio.01223-19.3 TABLE S3 Primers used in this study. Download Table S3, PDF file, 0.1 MB. Copyright © 2019 Frantz et al. 2019 Frantz et al. This content is distributed under the terms of the Creative Commons Attribution 4.0 International license.

### Construction of rli32 deletion mutant.

The chromosomal deletion mutant was constructed by generating the 5′ flanking region (with primers rli32-1-for and rli32-2-rev) and the 3′ flanking region (with primers rli32-3-for and rli32-4-rev) of rli32. The primer sequences used to generate the isogenic mutants are presented in Table S1. The PCR fragments were purified and ligated into temperature-sensitive suicide vector pAUL-A. Plasmid DNA of pAUL-A bearing the fragments were transformed in L. monocytogenes to generate the chromosomal deletion mutants as described previously (58).

### Construction of rli32 expression vectors.

To produce rli32 overexpression vector, the sRNA was amplified from chromosomal DNA and fused with the hemolysin (*hly*) promoter and *hly* terminator of L. monocytogenes. The primers used to generate the flanking regions are shown in the supplemental material (Table S3 [restriction sites are underlined]). The purified PCR fragments containing the *hly* promoter upstream and the *hly* terminator downstream of rli32 were digested with either SalI or XmaI and ligated into the SalI and XmaI sites of multicopy vector pERL3, which was used for transformation into electrocompetent L. monocytogenes cells. Similarly, we generated a vector that produced rli32 under the control of its native promoter. The transformed clones were selected on BHI agar plates containing 5 μg/ml erythromycin.

### RNA extraction, cDNA synthesis, and RNA-Seq; dephosphorylation of RNA molecules; and generation of *in vitro*-transcribed RNA.

Eukaryotic RNA was isolated from HEK293 cells and bone marrow-derived macrophages (BMDM) using an miRNeasy minikit (Qiagen). To isolate bacterial cytosolic RNA, additional steps were performed to lyse the bacterial cells before the addition of QIAzol (30). Details of RNA extraction, sequencing, and dephosphorylation and cDNA synthesis and generation of *in vitro*-transcribed RNA are provided in Data Set S1 in the supplemental material.

### Cell lines and transfection.

BMDM were isolated from bone marrow of C57BL/6 mouse strains and used after 6 days of cultivation in RPMI 1640 medium (Thermo Fisher Scientific) supplemented with 10% fetal calf serum (FCS) (Biochrom GmbH), 100 μg/ml streptomycin and100 U/ml penicillin (Thermo Fisher Scientific), and 30% supernatant from the L929 cell line. Cells of the HEK293 cell line (human embryonic kidney cells) were maintained in DMEM (Dulbecco’s modified Eagle medium) (Thermo Fisher Scientific) supplemented with 10% FCS. HEK293 RIG-I and MDA5^−/−^ cells were kindly provided by Veit Hornung (59). BMDM cells were transfected with sec-RNA, MV-RNA, and cytosolic RNA (100 ng/10^6^ cells) or with IVT-RNA (50 ng/10^6^ cells), complexed with Lipofectamine 2000 according to the manufacturer’s protocol (Thermo Fisher Scientific). HEK293 cells were transfected with sec-RNA, MV-RNA, and cytosolic RNA (100 ng/10^5^ cells). Transfection of IVT-RNA was performed by using 50 ng RNA. The transfection efficiency was determined by qRT-PCR.

For RIG-I activation assay, A549 cells (lung carcinoma cells from human) were maintained in DMEM supplemented with 10% FCS, 50 U/ml penicillin, and 50 μg/ml streptomycin at 37°C and 5% CO_2_. For virus titration, MDCK-II cells (Madin-Darby canine kidney cells) were maintained in DMEM supplemented with 100 U/ml penicillin, 100 μg/ml streptomycin, and 10% FCS. All cells were incubated at 37°C in the presence of 5% CO_2_.

### Cell culture infection experiment.

BMDM, P388D1 macrophages, and HEK293 cells were infected with L. monocytogenes as described previously (30). Briefly, the eukaryotic cell were cultured in RPMI 1640 (Thermo Fisher Scientific) supplemented with 10% FCS in 24-well tissue culture plates in a humidified incubator. The cells were grown to about 80% confluence.

Bacteria were added to the cell monolayer at a multiplicity of infection (MOI) of 10 bacteria per eukaryotic cell. Infection was carried out for 45 min in macrophages or for 1 h in HEK293 cells, followed by the addition of fresh medium containing 50 μg/ml of gentamicin to kill extracellular bacteria. Intracellular growth assays were performed at different time points (e.g., 4, 8, or 24 h postinfection). At each step, the plates were washed extensively with phosphate-buffered saline. For IFN inhibition, the macrophages were treated 30 min prior to infection with 1 μM or 10 μM ruxolitinib (Selleck Chemicals) (42) or were treated with dimethyl sulfoxide (DMSO) (control).

### Data availability.

Sequences originating from this study have been deposited in the European Nucleotide Archive. All the sequencing data are available under project identifier PRJEB21884.
