# Supplementary material for: The secRNome of Listeria monocytogenes Harbors Small Noncoding RNAs That Are Potent Inducers of Beta Interferon
Source: mBio. 2019 Oct 8;10(5):e01223-19. doi: 10.1128/mBio.01223-19 (PMC6786865; doi:10.1128/mBio.01223-19)
Supplement: DATA SET S1 [file mBio.01223-19-sd001.docx]

**Supplementary Information for**

**The secRNome of *Listeria monocytogenes* harbors small non-coding RNAs that are potent inducers of IFN-β**

Renate Frantz^1¶^, Lisa Teubner^1¶^, Tilman Schultze^1^, Luigi La Pietra^1^, Christin Müller^2^, Konrad Gwozdzinski^1^, Helena Pillich^1^, Torsten Hain^1^, Michaela Weber-Gerlach^3^, Georgios-Dimitrios Panagiotidis^3^, Ahmed Mostafa^2*^, Friedemann Weber^3^, Manfred Rohde^4^, Stephan Pleschka^2^, Trinad Chakraborty^1#^, Mobarak Abu Mraheil^1#^

Corresponding authors: Mobarak Abu Mraheil and Trinad Chakraborty

E-mail: mobarak.mraheil@mikrobio.med.uni-giessen.de

E-mail: trinad.chakraborty@mikrobio.med.uni-giessen.de

**This file includes:**

Figures S1-S13

Supplemental Materials and Methods

References for SI reference citations

**
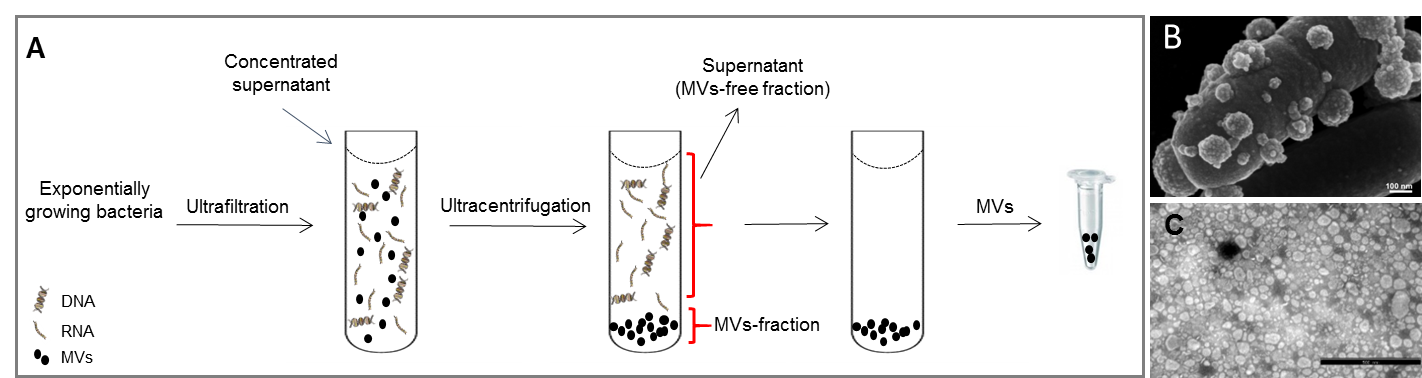
**

**Figure S1.** Isolation of RNA from the supernatant and membrane vesicles.

(A) Schematic representation of the established method for co-isolation of membrane vesicles (MVs) and sec-RNA released by *L. monocytogenes*. Sec-RNA and MVs were isolated from exponentially grown cultures in defined minimal medium. Following centrifugation and sterile filtration, the supernatant was concentrated 66-fold by ultrafiltration. Ultracentrifugation was then used to separate MVs in the pellet from secreted nucleic acids in the supernatant. Sec-RNA was precipitated from the supernatant as described in material and methods. In order to extract MVs-associated RNA, 50 µg of purified MVs were first subjected to RNase treatment prior to isolation of intravesicular RNA. The cytosolic RNA was isolated from the bacterial cells collected by centrifugation.

(B) MVs shown as spherical blebs surrounding the bacteria were visualized by SEM.

(C) Visualization of isolated MVs using TEM.

**
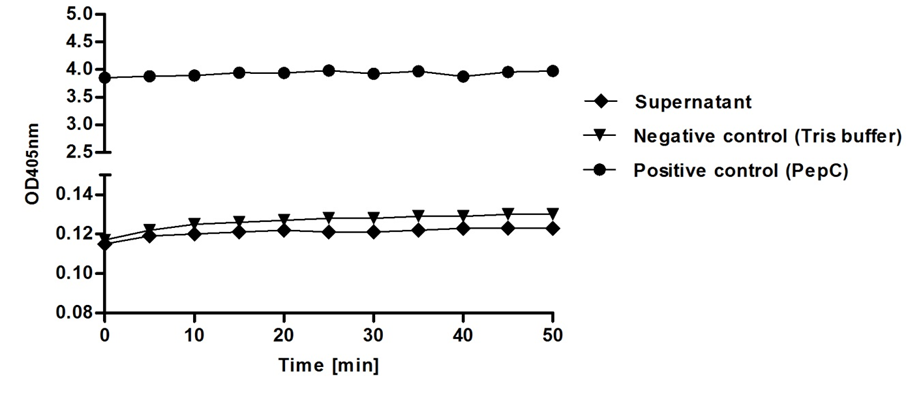
**

**Figure S2.** Evaluation of potential cell lysis by aminopeptidase C assay. The supernatant from exponentially grown *L. monocytogenes* cultures in defined minimal medium was tested. Positive control (PepC) = purified aminopeptidase C from *Aeromonas proteolytica*; negative control was Tris buffer. The results represent one of three independent experiments. The aminopeptidase C activity was assayed immediately by measuring the absorption at 405_nm_ for 50 minutes.

**
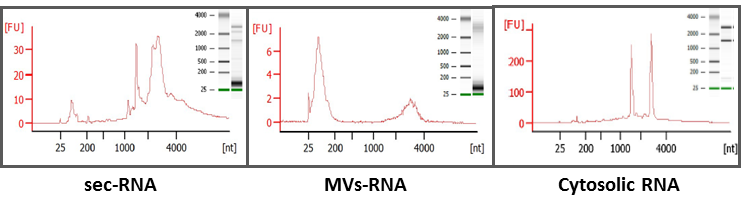
**

**Figure S3.** Size distribution of RNAs in sec-RNA, MVs-RNA and cytosolic RNA fractions. Bioanalyzer electropherograms (RNA 6000 Nano Chip) indicating an enrichment of short RNAs in sec-RNA and MVs-RNA isolated from *L. monocytogenes* wild type.

**
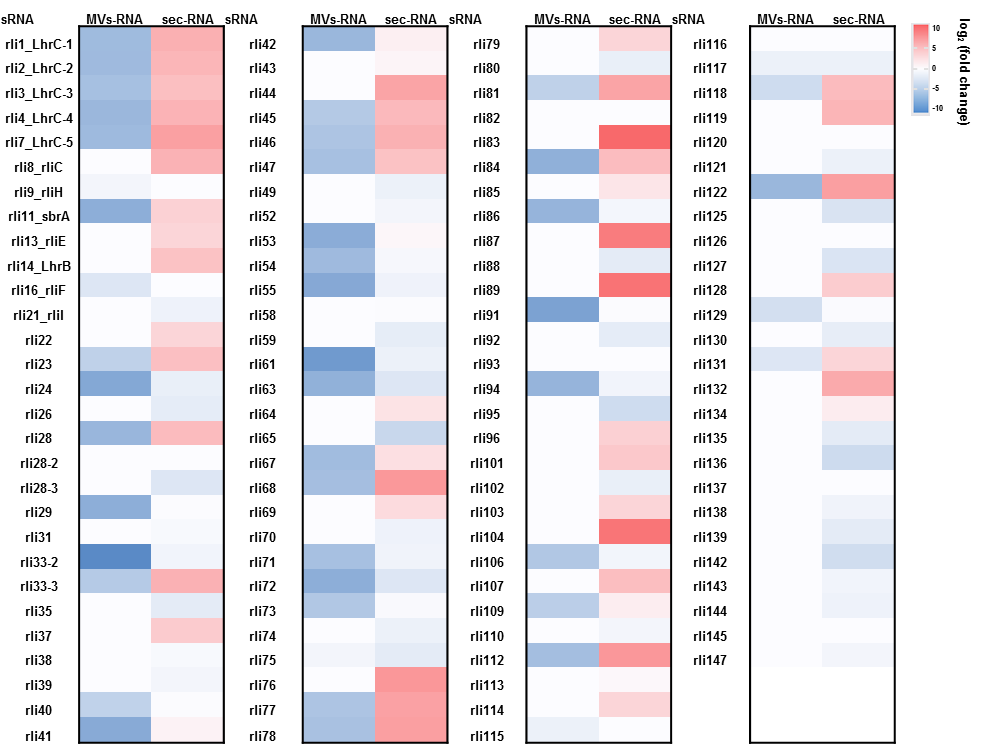
**

**Figure S4.** Heat map depiction of the relative sRNA amounts in MVs-RNA and sec-RNA as compared to cytosolic RNA in *L. monocytogenes* wild type.

**
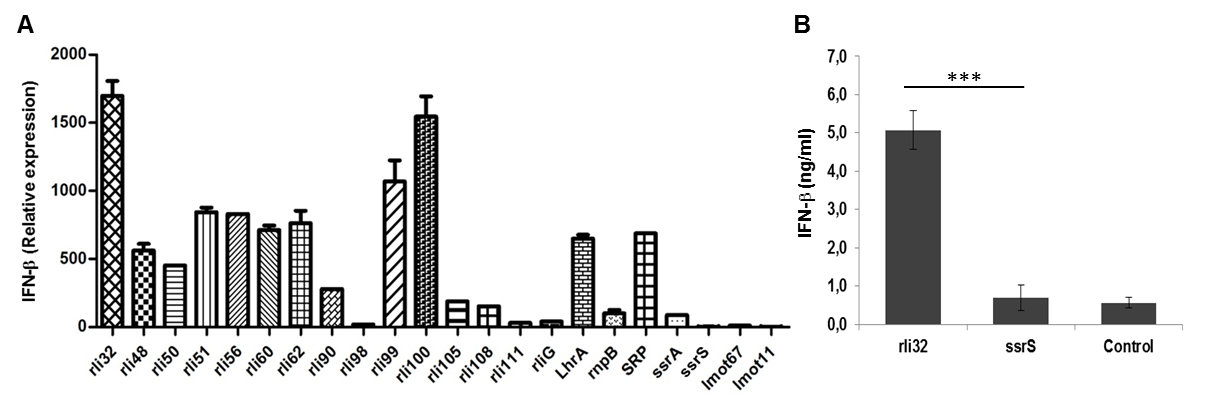
**

**Figure S5.** IFN-β induction by *in-vitro* transcribed sRNAs. (A) IFN-β induction in HEK293 cells by individual sRNAs detected by RNA-Seq in sec-RNA and MVs-RNA. HEK293 cells were transfected with different IVT sRNA molecules (50 ng/10^5^ cells). The IFN-β induction was assessed by qRT-PCR 24 h after transfection. (B) IFN-β protein amounts (ng/ml) determined after transfection of rli32 and ssrS in HEK-Blue IFN-α/β cells (InvivoGen). Cells treated with the transfection reagent (lipofectamine) only were used as control (***P < 0.001).

**
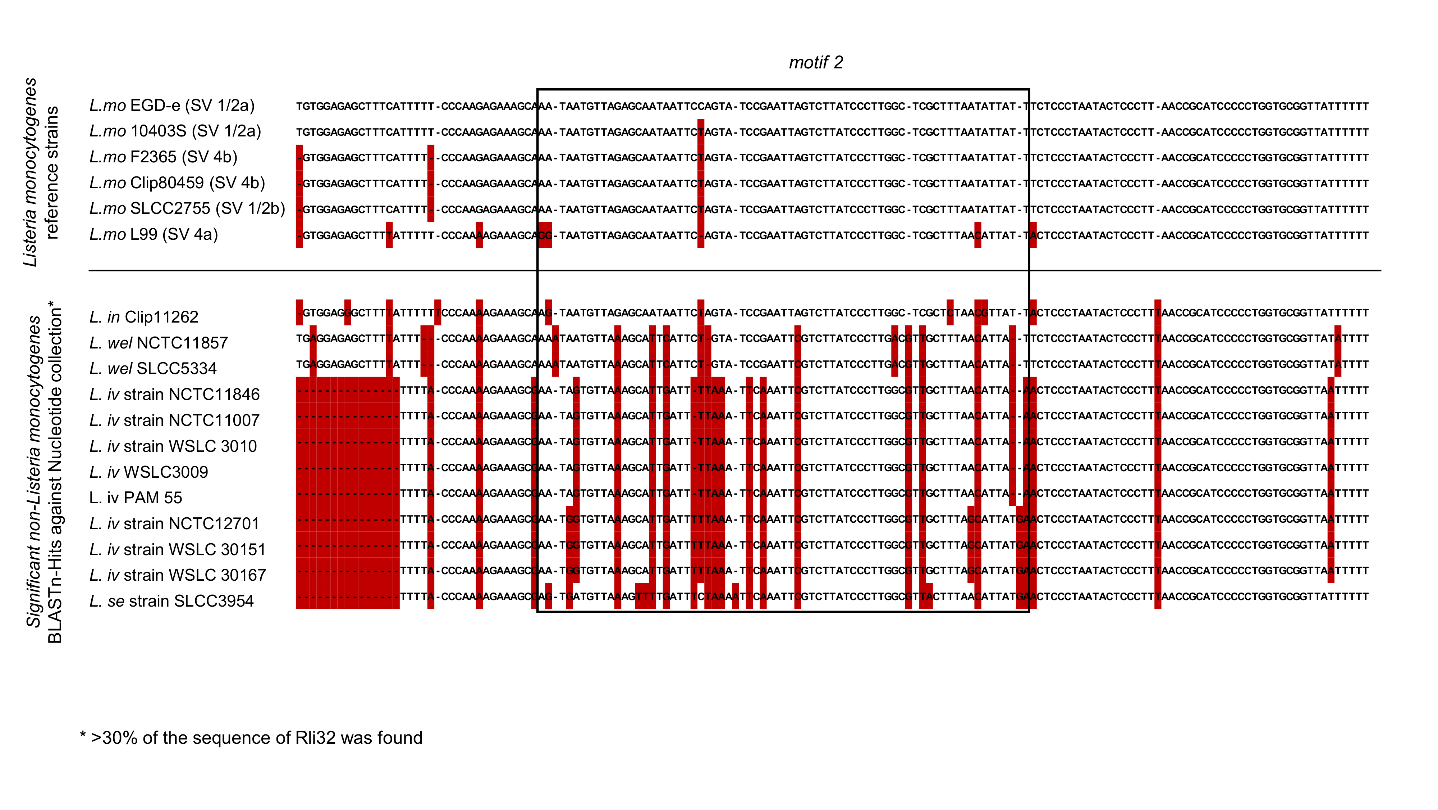
**

**Figure S6.** Multiple sequence alignment of rli32. Six representative *L. monocytogenes* strains are depicted. Red colored nucleotides illustrate differences in rli32 sequence as compares to *L. monocytogenes* EGD-e used in this study. *L. in. = Listeria innocua, L. wel. = Listeria welshimeri, L. iv. = Listeria ivanovii, L. se. = Listeria seeligeri.* * >30% of the sequence of rli32 was found.

**
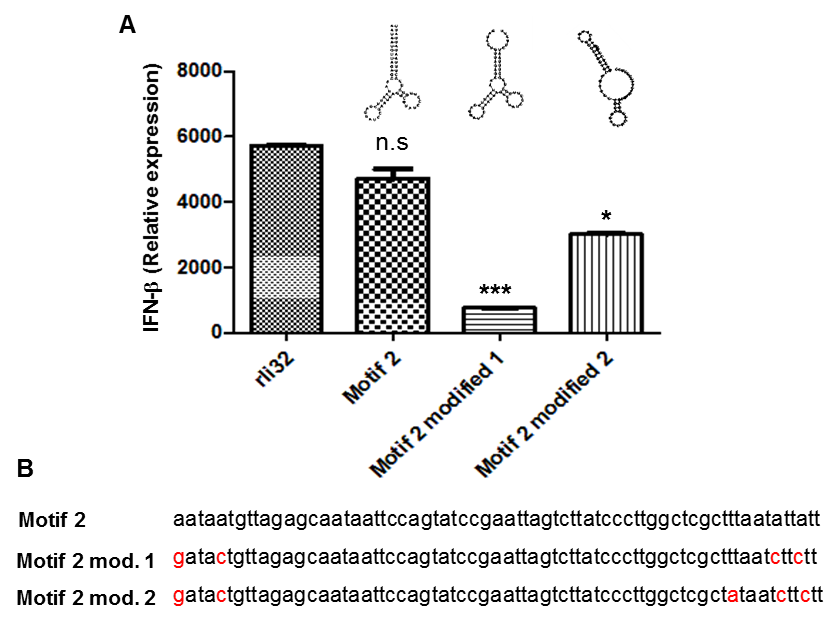
**

**Figure S7.** IFN-β induction properties of two different modified variants of motif 2. (A) Ability of modified variants of motif 2 to induce IFN-β response after transfection in macrophages. Cells treated only with the transfection reagent (lipofectamine) only were used as control. (B) Sequence alteration that cause the structural modifications of motif 2 introduced by PCR-in vitro mutagenesis. Data are presented as mean ± SD of three experiments (ns = non-significant, *P < 0.05, ***P < 0.001).

**
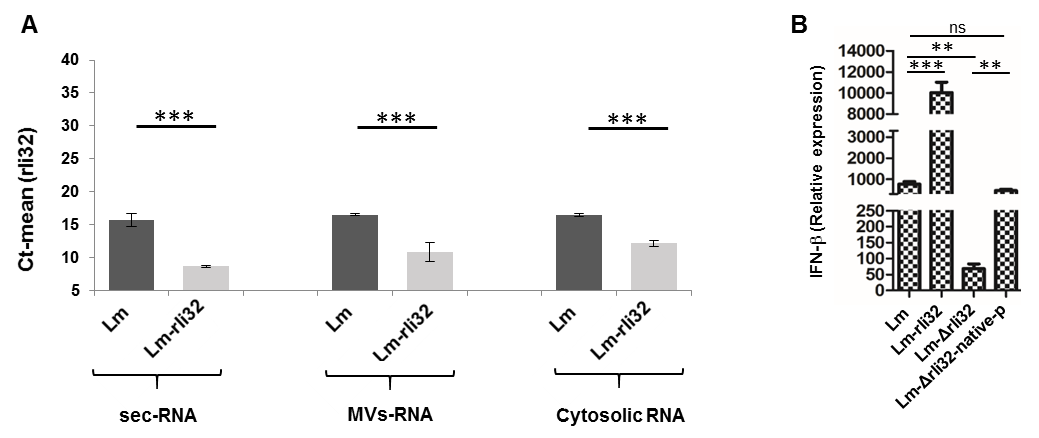
**

**Figure S8.** (A) Increased amounts of rli32 in the overproducing strain**.** Comparison of the amounts of rli32 in sec-RNA, MVs-RNA and cytosolic RNA isolated from the wild type *L. monocytogenes* (Lm) and the rli32 overproducing *L. monocytogenes* strain (Lm-rli32) using qRT PCR. Differences in the mean cycle threshold (Ct) are depicted.

(B) Comparison of IFN-β induction capabilities of sec-RNA isolated from Lm-rli32, Lm-Δrli32 and complemented Lm-Δrli32 that produces rli32 under the control of its native promoter (Lm-Δrli32-native-p) after transfection in macrophages. Cells treated only with the transfection reagent (lipofectamine) only were used as control. Data are presented as mean ± SD of three experiments (ns = non-significant, **P < 0.01, ***P < 0.001).


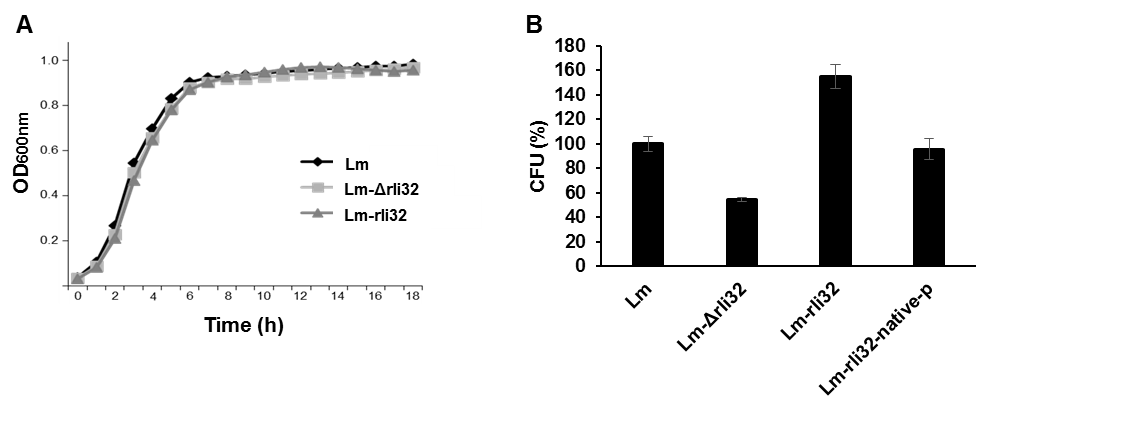


**Figure S9.** (A) Extracellular growth of Lm, Lm-Δrli32 and Lm-rli32 in BHI. *In-vitro* growth of the parental strain *L. monocytogenes,* its isogenic Δrli32 deletion mutant and the rli32 overproducing strain at 37°C in BHI broth. Values represent one of three independent experiments.(B) Comparison of intracellular growth capabilities of Lm, Lm-rli32, Lm-Δrli32 and complemented Lm-Δrli32 that produces rli32 under the control of its native promoter (Lm-Δrli32-native-p).

**Figure S10.** Heat map of differentially expressed mRNAs in (A) sec-RNA, (B) MVs-RNA and (C) cytosolic RNA in the overexpression strain (Lm-rli32) and deletion mutant (Lm-Δrli32) as compared to the wild type (Lm). More information are available in table S2.

**
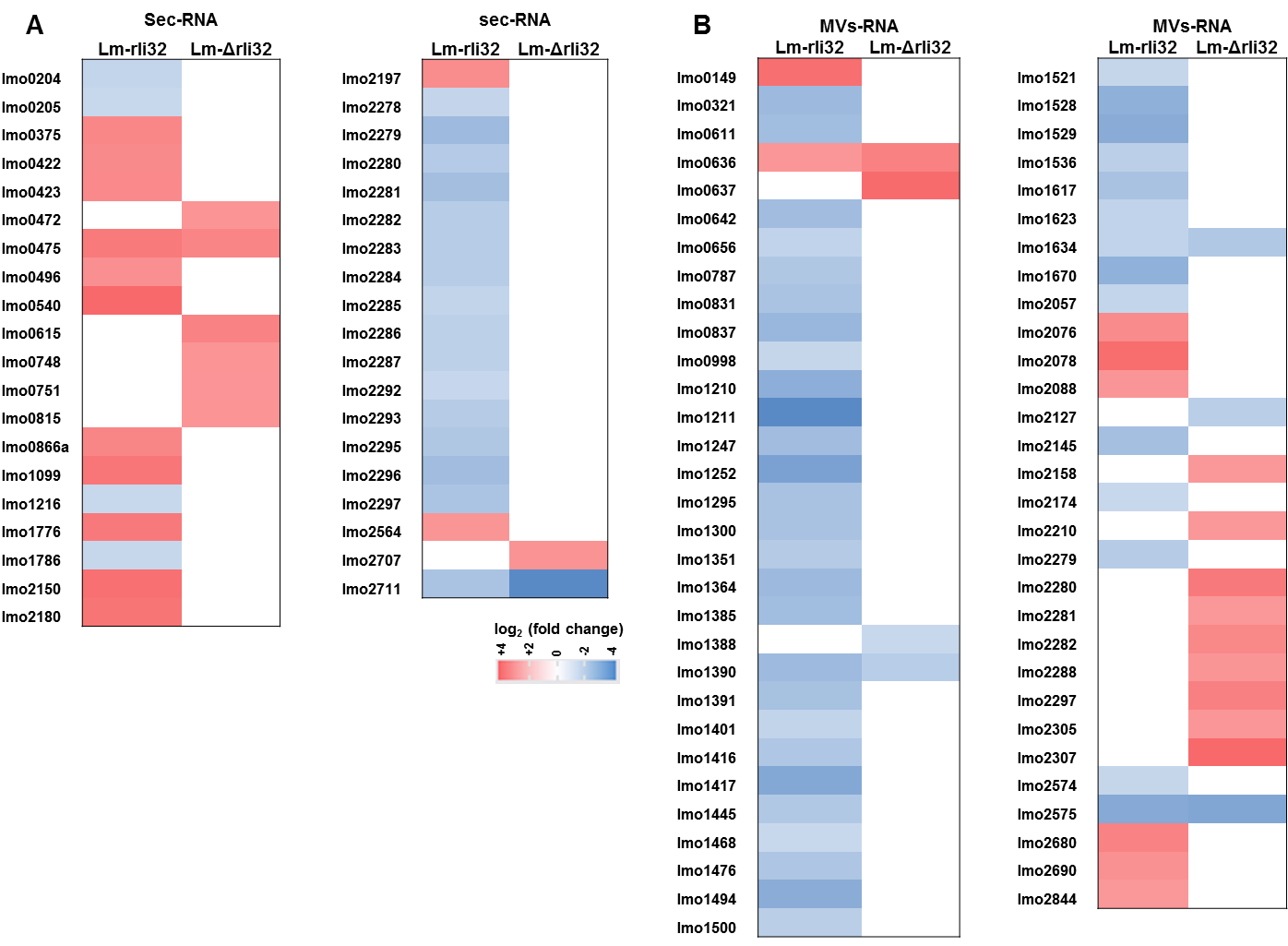
**

**
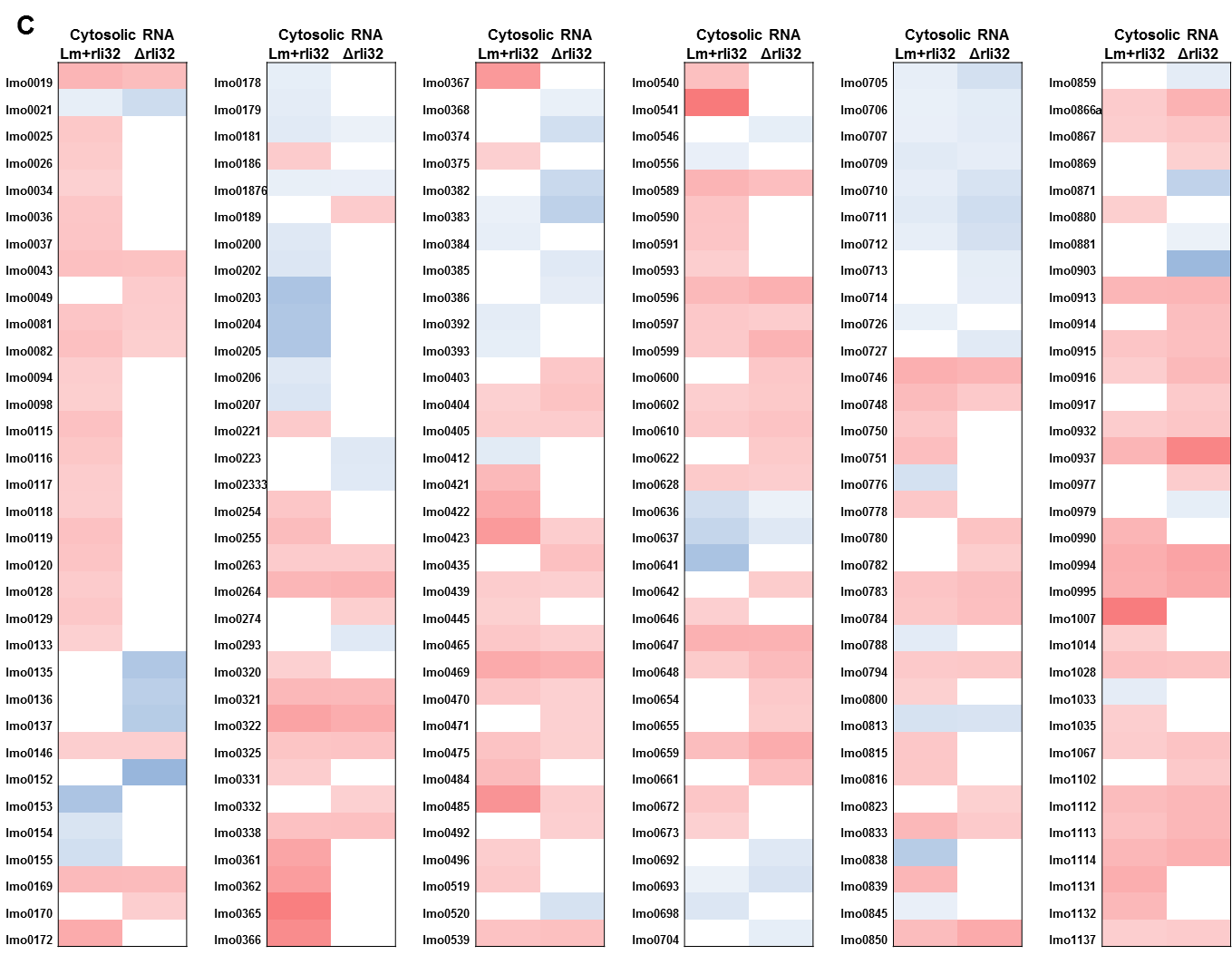
**

**
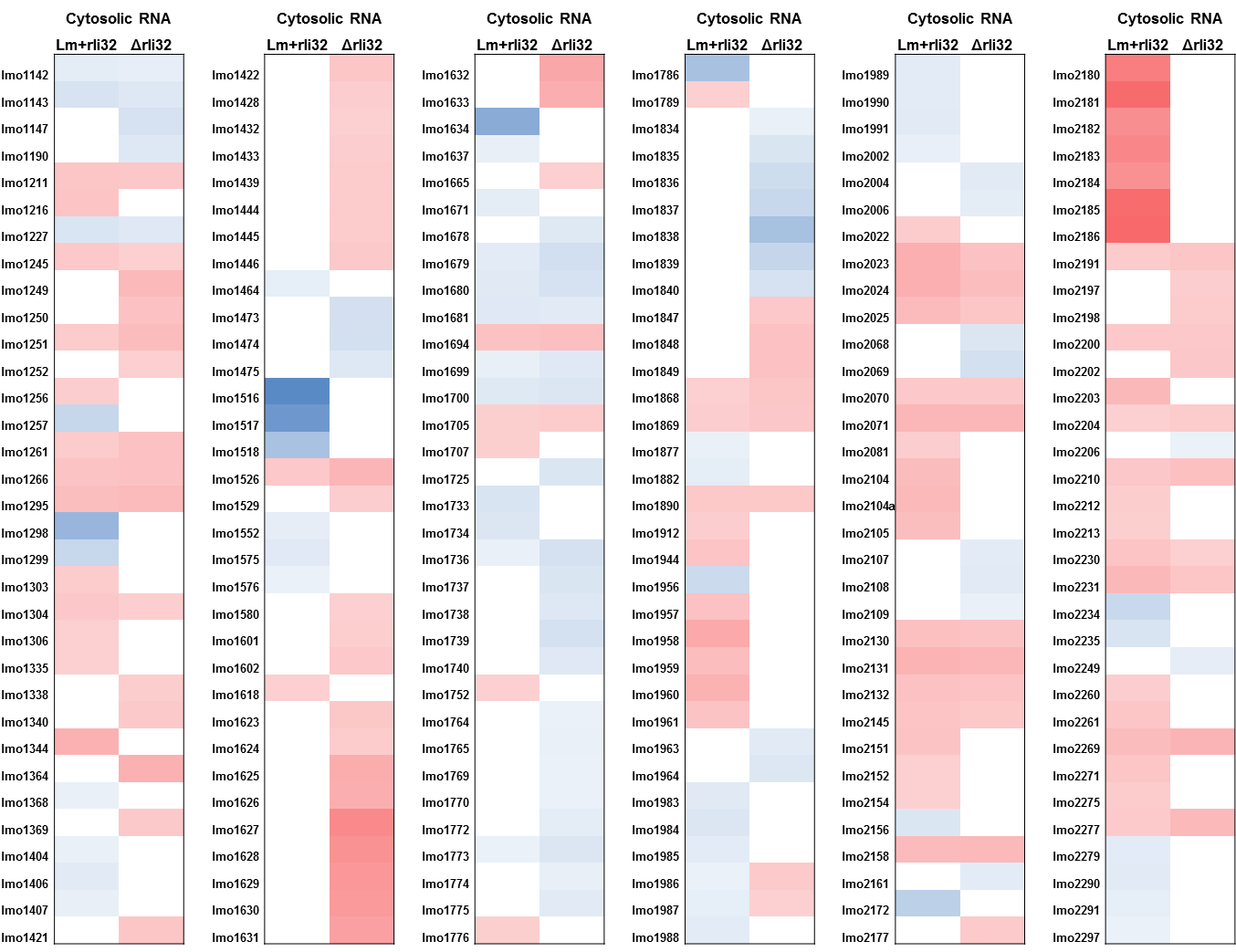
**

**
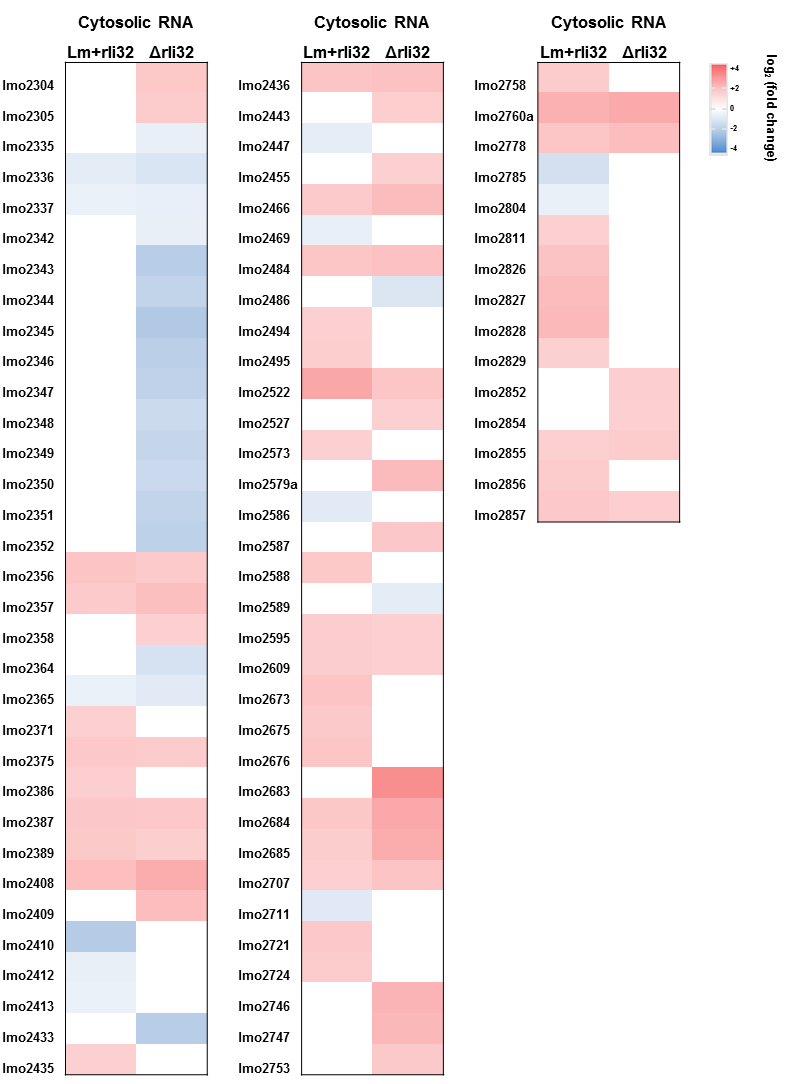
**

**
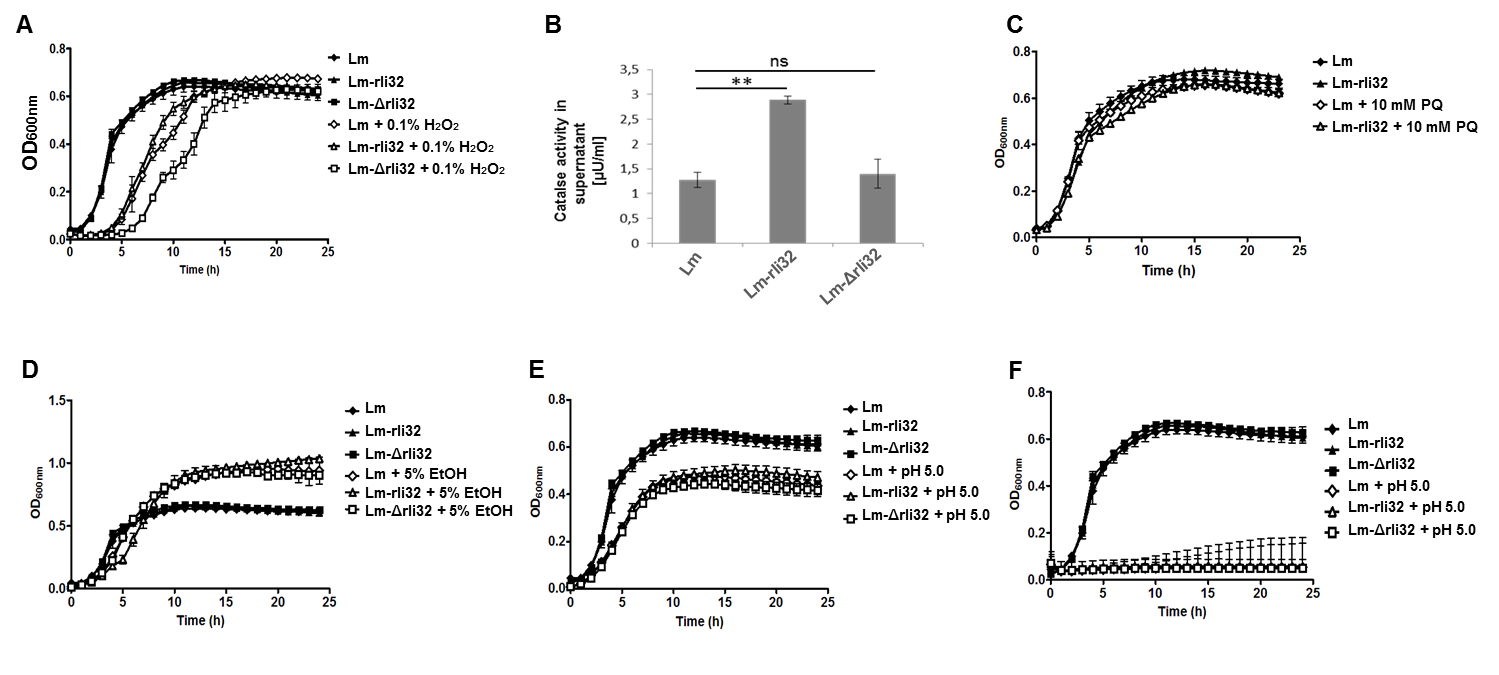
**

**Figure S11.** The impact of different stress conditions on the growth of isogenic parental strain (Lm), rli32 overproducing strain (Lm-rli32) and rli32 deletion mutant (Lm-Δrli32). (A) Impact of 0.1% H_2_O_2_. (B) Catalase production in the presence of 0.15% H_2_O_2_. (B) Impact of 10 mM paraquat (PQ). (D) Impact of 5% ethanol. (E-F) Impact of acidic pH values (5.0 and 2.5). Data are presented as mean ± SD of three experiments (ns = non-significant, **P < 0.01).

**
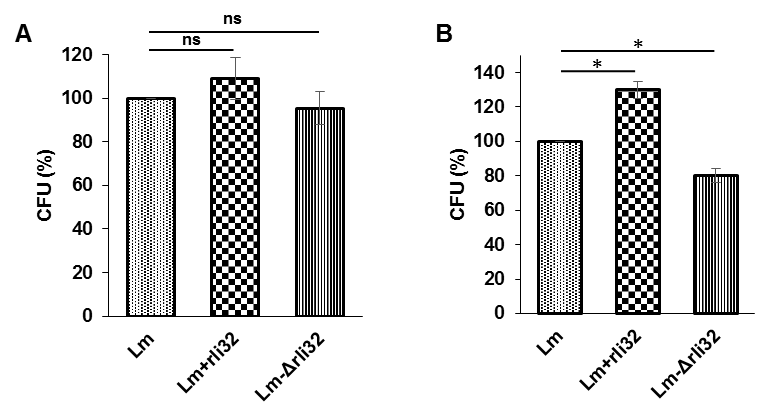
**

**Figure S12.** Impact of RIG-I on rli32 dependent intracellular growth.

(A) Growth of Lm, Lm-rli32 and Lm-Δrli32 in HEK293 RIG-I^-/-^ deficient HEK293 cells and (B) wild type HEK293 cells 24 h p.i.. Data are presented as mean ± SD of three experiments (ns = non-significant, *P < 0.05).

**Table S1. RNA-Seq data of sec-RNA, MVs-RNA and cytosolic RNA.**

The data sets represent three cDNA libraries originate from three biological replicates (3x sec-RNA and 3x MVs-RNA). The sequenced reads were aligned to the genome of *L. monocytogenes* EGD-e using ‘CLC Genomic Workbench’ version 8.5.1.

**Table S2.** Differentially expressed mRNAs in sec-RNA, MVs-RNA and cytosolic RNA of the overexpression strain (Lm-rli32) and deletion mutant (Lm-Δrli32) as compared to the wild type (Lm). Supplementary information for figure S8.

**Table S3. Primers used in this study.**

**Supplemental Materials and Methods**

**Aminopeptidase assay.**

To exclude the possibility that the sec-RNA is derived through cell lysis, we examined the presence of the strictly cytoplasmic enzyme aminopeptidase C (PepC) in the supernatant.10 µl supernatant from exponentially grown *L. monocytogenes* cultures were added to 190 µl 20 mM Tris-HCl, pH 7.4 in microtiter-plates. Afterwards 2 µl 200 mM L-arginine-p-nitroanilide (Sigma) solved in 20 mM Tris-HCl, pH 7.4 were added to the samples. Five units of purified aminopeptidase C from *Aeromonas proteolytica* (Sigma) solved in 20 mM Tris-HCl, pH 7.4 were used as a positive control instead of the supernatant. The aminopeptidase C activity was assayed immediately by measuring the absorption at 405 nm for 1 hour. Negative control (Tris buffer) was 20 mM Tris-HCl, pH 7.4.

**Purification of membrane vesicles.**

MVs were purified from supernatants of *L. monocytogenes*. For this purpose 2 liters (4 x 500 ml) *L. monocytogenes* cultures were grown in (MM) at 37°C in an orbital shaking incubator at 180 rpm until reaching exponential phase (OD_600nm_ = 1.0). The grown bacteria were pelleted at 6,000 x g for 15 min and the supernatant was sterile-filtered (Millipore Express PLUS Membrane Filter, PES, 0.22 µm) to remove residual bacteria. Afterwards the bacteria free supernatant was concentrated by ultrafiltration using KrosFlo Research II TFF using a 30 kDa hollow fiber membrane (Spectrum Labs) to a final volume of 30 ml. The resulting filtrate (30 ml) was subjected to ultracentrifugation at 150,000 × g for 3 h and 4°C in a SW41 Ti rotor (Beckman) to separate MVs fraction (MVs pellet) and MVs-free fraction (MVs-free supernatant). Subsequently the MVs-free supernatant was collected for the extraction of sec-RNA. The MVs included in the pellet were resuspended in 300 µl PBS (Dulbecco’s Phosphate Buffered Saline, Biochrom GmbH), sterile filtered (Millex-GV Syringe Filter Unit, PVDF, 0.22 µm) and stored at -80°C until use. The amount of isolated MVs was quantified by protein concentration measurement using Bradford protein assay.

**Field-emission scanning and transmission electron microscopy.**

The overnight bacterial culture was fixed with 5% formaldehyde and 1% glutaraldehyde in growth medium and dehydrated in a graded series of acetone (10%, 30%, 50%, 70%, 90% and twice 100%) on ice for 10 min each. Samples were then subjected to critical-point drying apparatus with liquid CO_2_ (CPD 030, Balzers, Liechtenstein). Dried samples were covered with a gold-palladium film by sputter coating (SCD 500, Balzers, Liechtenstein) before examination by field-emission scanning electron microscope (FE-SEM) Zeiss Merlin (Carl Zeiss, Germany) with an accelerating voltage of 5 kV. Images were taken by Inlens SE-detector and High efficiency SE-detector in a 50:50 ratio.

For negative-staining transmission electron microscopy (TEM) the MVs sample were applied on the formvar coated copper grids, negatively stained with 1% ammonium heptamolybdate and subsequently decanted. Stained grids were allowed to air dry and then the sample was observed and imaged using a Zeiss EM900 transmission electron microscope (Zeiss NTS, Oberkochen, Germany).

**Isolation of cytosolic RNA.**

Eukaryotic RNA was isolated from HEK293 cells and bone marrow-derived macrophages (BMDM) using the miRNeasy Mini Kit (Qiagen). Briefly, the cells were lysed by QIAzol, mixed gently and incubated for 5 min at room temperature (RT). Subsequently, 0.2 volume of chloroform was added to the homogenate. After incubation for 2 min at RT the sample was centrifuged at 16,000 x g at 4°C for 15 min. The upper aqueous phase, containing RNA, was transferred into a new collection tube and 1.5 volumes of 100% ethanol was added and mixed thoroughly. The probes containing RNA and ethanol were transferred into columns supplied with the miRNeasy Mini Kit (Qiagen) and treated according to the manual including an on-column DNase digestion (RNase-Free DNase, Qiagen). RNA was eluted by RNase-free water and stored at -80°C until needed. The quantity of the isolated cytosolic RNA was determined using Qubit RNA BR Assay Kit (Thermo Fisher Scientific), and the quality was assessed using Nano-chips for Agilent 2100 Bioanalyzer (Agilent Technologies, Diegem, Belgium).

The cytosolic RNA was isolated from the bacterial cells collected by centrifugation (Fig. S1). To isolate bacterial cytosolic RNA additional steps were performed to lyse the bacterial cells before the addition of QIAzol (3). Briefly, the collected bacterial pellets were washed with SET buffer [50 mM NaCl, 5 mM EDTA and 30 mM Tris-HCl (pH 7.0)] containing 10% sodium dodecyl sulfate. After centrifugation at 16,000 g for 3 min, the pellets were resuspended into 0.1 ml 50 mM Tris-HCl (pH 6.5) containing 50 mg/ml lysozyme (Sigma), 25 U of mutanolysin (Sigma), 40 U of SUPERase (Ambion), 0.2 mg of proteinase K (Ambion) and incubated at 37°C for 45 min at 350 rpm. After lysis of the bacterial cells cytosolic RNA was purified using the miRNeasy Mini Kit (Qiagen) (3).

**Extraction of MVs-RNA.**

The extraction of RNA present in MVs (MVs-RNA) was performed by using 50 µg MVs. Prior to extraction, MVs were treated with 2.5 µg RNase (Roche) to digest RNA bound to the outer surface of MVs. Subsequently, MVs were mixed with 1.5 volume Roti-Aqua-Phenol/Chloroform/Isoamylalcohol (25:24:1, pH = 4.5-5.0 for RNA isolation). The RNA was precipitated with 0.1 volume of 3 M NaOAc (pH 4.8-5.2) and 2 volumes of absolute ethanol (Sigma-Aldrich) by centrifugation at 16,000 x g for 15 min at 4°C. Extracted RNAs were treated with DNase I (Ambion) to remove DNA contamination. The RNA content of MVs was determined by Qubit RNA BR Assay Kit (Thermo Fisher Scientific). The size distribution of MVs-RNA was analyzed with the Agilent RNA 6000 Nano Kit using Agilent 2100 Bioanalyzer (Agilent Technologies, Diegem, Belgium).

**Isolation of sec-RNA.**

RNA that occur in the MVs-free supernatant was precipitated in the presence of 1 volume of ethanol absolute (Sigma-Aldrich) over night at -20°C. Afterwards, the RNA was collected by centrifugation at 4°C for 30 min at 6,000 x g. The extracted RNA was purified using the miRNeasy Mini Kit (QIAGEN). The RNA concentration and size distribution was determined as mentioned for MVs-RNA.

**cDNA synthesis and RNA-Sequencing.**

First, the RNA samples were treated with RNA 5' Polyphosphatase (Epicentre). Oligonucleotide adapters were ligated to the 5' and 3' ends of the RNA samples. First-strand cDNA synthesis was performed using M-MLV reverse transcriptase and the 3’ adapter as a primer. The resulting cDNAs were amplified by PCR using a high fidelity DNA polymerase. The cDNA was purified using the Agencourt AMPure XP kit (Beckman Coulter Genomics). The yield and size distribution of the amplified cDNA were assessed with Agilent DNA High Sensitivity Kit (Agilent, Santa Clara, CA, USA). Libraries were then diluted to 4 nM, pooled, denatured and further diluted to 10 pM. Sequencing was carried out on the MiSeq using v2 chemistry (Illumina, San Diego, CA, USA).

Adapter sequences were removed from sequencing data using cutadapt 1 (DOI:10.14806/ej.17.1.200). CLC Genomics workbench 8.5.1 (CLC bio, Qiagen) was then utilized to align processed reads to the reference (NC_003210) and count reads. As parameters for the mapping, a mismatch cost of 2, an insertion cost of 3, a deletion cost of 3 along with a threshold for the length fraction of 0.8 and a required similarity fraction of 0.8 were chosen. The counting was carried out in a strand-specific manner.

Sequences originating from this study are deposited in “European Nucleotide Archive”. All the sequencing data is available under the project PRJEB21884.

**Generation of *in-vitro* transcribed RNA.**

For generation of DNA-template-dependent *in-vitro* transcribed RNA, the T7 RNA polymerase promoter region was hybridized with the forward primer (Table S3). The generated DNA was used as a template for the *in-vitro* transcription (IVT) reaction by using a commercial *in-vitro* T7 transcription kit (New England Biolabs). The respective RNAs were analyzed on denaturing polyacrylamide gel electrophoresis (7 M Urea, 10% PAGE) and stained using ethidium bromide. RNA bands of the expected size were cut out on a UV table. The gel slices were fragmented and the RNA was eluted using an elution buffer (0.5 M NaOAc (pH 5.0), 1mM EDTA (pH 8.0), 2.5% v/v phenol). Subsequently, the eluted RNA was purified by phenol/chloroform (1.5 volume Roti-Aqua-Phenol/Chloroform/Isoamylalcohol (25:24:1, pH = 4.5-5)) and precipitated with ethanol in the presence of 3 M NaOAc pH 4.8-5.2.

**Dephosphorylation of RNA molecules.**

To remove 5`-triphosphates from IVT RNA, sec-RNA and MVs-RNA 1 µg of each was incubated with 10 units of alkaline phosphatase (CIAP = calf intestinal alkaline phosphatase; New England BioLabs) for 60 min at 37°C. The dephosphorylated RNA was purified by Roti-Aqua-Phenol/Chloroform/Isoamylalcohol extraction, followed by ethanol precipitation in the presence of 3 M NaOAc pH 4.8-5.2.

**Quantitative RT-PCR (qRT-PCR).**

DNase I (RNase-free DNase Set, Qiagen) treated RNA (500 ng) was reverse transcribed into cDNA by using SuperScript II Reverse Transcriptase (Thermo Fisher Scientific). The cDNA amplification was performed with QuantiTect SYBR Green PCR Kit (Qiagen) on a StepOnePlus Real-Time PCR System (Applied Biosystems). The primers used to amplify the cDNA were custom-made QuantiTect Primer Assays (Qiagen). The cDNA amplification (human) was performed by the primers: IFN-β (Hs_IFNB1_1_SG QuantiTect Primer Assay (QT00203763)) and the reference gene PPIA (peptidylprolyl isomerase A (cyclophilin A)) (Hs_PPIA_1_SG QuantiTect Primer Assay (QT00052311)). For amplification of cDNA from mice cells the primers for IFN-β (Mm_Ifnb1_1_SG QuantiTect Primer Assay (QT00249662)) and the reference gene Rplp: (ribosomal protein, large, P0) Mm_rplp0_1_SG QuantiTect Primer Assay (QT00249375) were used.

For each indicated gene, as well as for the reference gene, a standard curve was generated to calculate the quantity of mRNA as a function of the Ct value. The expression level of each gene was calculated by normalizing its mRNA quantity to the quantity of the reference gene mRNA for the same sample using the equation described by Pfaffl (1). The baseline was set automatically and the threshold was set to 0.5.

**Infection of HEK293 cells with A/Puerto Rico/8/34 (PR8, H1N1) and Virus titration.**

HEK293 cells, transfected with rli32 and ssrS or control non-transfected, were washed with PBS^++^ (1x phosphate-buffered saline containing 1 mM MgCl_2_, 0.9 mM CaCl_2_) and were infected with the influenza virus strain A/Puerto Rico/8/34 (PR8, H1N1) at a multiplicity of infection (MOI) = 0.1 in PBS^++^/BA/Pen-strep (PBS^++^ containing 0.2% bovine albumin (PAA, Germany), 100 U/ml penicillin, and 0.1 mg/ml streptomycin) for 1 h at room temperature (RT). Afterwards, the inoculum was aspirated, and the cells were incubated with DMEM/BA medium (DMEM containing 0.2% BA, 100 U/ml penicillin, and 0.1 mg/ml streptomycin) at 37°C, 5% CO_2_. After for 24 h, the supernatants (virus samples) were collected to determine the virus titers. The MDCK-II cells were cultured in 96 well plates over night at 37°C, 5 % CO_2_ to about 90% confluency. Then, cells were washed once with PBS^++^ and infected with 50 μl of 10 fold serially diluted virus samples in PBS^++^/BA/Pen-strep for 1 h at RT. The inoculum was aspirated, 150 μl titration medium (1x MEM containing P/S, 0.2% BSA, 1.25% Avicell (FMC BioPolymer, Belgium), 1% DEAE-Dextran, 0.3% NaHCO_3_, and 1 µg/ml TPCK-treated trypsin) was added and cells were further incubated at 37°C, 5% CO_2_ for 24 h.

To detect foci of infected cells resulting from an infectious particle, cells were fixed and permeabilized with 150 μl fixing solution (4% paraformaldehyde, PFA, Roth, Germany) and 1% Triton X-100 (Roth, Germany) in PBS^++^ and stored at 4°C for 1 h. After this incubation period, the solution was discarded and cells were washed 3x with washing buffer (PBS^++^/0.05% Tween20 (Roth, Germany)). Next, the cells were incubated with 50 μl/well primary antibody (mouse anti-influenza A virus nucleoprotein mAb, S. Ludwig. Münster, Germany) diluted 1:100 in PBS^++^ containing 3% BSA (PAN Biotech, Germany) for 1 h at RT. The cells were washed 3x with washing buffer and further incubated with 50 μl/well secondary antibody (goat anti-mouse HRP-antibody, Santa Cruz, USA) diluted 1:1000 in PBS^++^ containing 3% BSA, for 1 h at RT. Next, cells were washed 3x with washing buffer and incubated with 40 μl/well “AEC” staining solution (SantaCruz, USA). After sufficient staining, the substrate was removed and cells were washed 2x with dH_2_O.

To detect and quantify foci (indicating a productive replication), the 96 well plates were scanned with a resolution of 1200 dpi using the Epson Perfection V500Photo scanner (Epson) and analyzed using “Photoshop” software package (Adobe, USA). Results represent the averages from three independent Experiments.

All experiments with infectious virus were performed according to German regulations for the propagation of influenza viruses. All experiments involving PR8 were performed in biosafety level 2 (BSL2) containment laboratories approved for such use by the local authorities of Giessen, Germany.

**RIG-I activation assay.**

To monitor RIG-I activation, A549 cells were transfected with 200 ng/ml of ssrS, rli32 or 5`-triphosphorylated double-stranded RNA (3pRNA; Invivogen) using Lipofectamine 2000 (Thermo Fisher Scientific). After 2 h, cells were subjected to RIG-I activation assay as previously described (2). Briefly, one part of the lysate was left untreated (input control) and the other part was subjected to limited protease digestion. Therefore, the sample was digested for 15 min with 0.2 µg/µl TPCK-treated trypsin (treated with N-tosyl-L-phenylalanyl chloromethyl ketone) (Sigma-Aldrich) at 37°C. The digestion was stopped by adding 4-fold sample buffer (200 mM Tris-HCl (pH 6.8), 8% SDS, 40% glycerol, 25% β-Mercaptoethanol, 0.4% Bromphenol Blue) and boiling for 5 min at 100°C. Samples were subjected to 12% SDS-PAGE (sodium dodecyl sulfate polyacrylamide gel electrophoresis) and Western Blot analysis using mouse monoclonal anti-RIG-I antibody (ALME-1, Adipogen). Band intensity was quantified using Image Lab 5.2.1 (BioRad) and activated RIG-I represents the ratio of trypsin-resistant RIG-I to undigested RIG-I.

**Catalase Activity Assay**

The highly sensitive and direct measurement of catalase activity was performed by using the Catalase Activity Assay Kit (ab83464) from Abcam. Bacterial cells were grown in defined minimal medium and harvested at late exponential phase by centrifugation at 15,000 x g for 20 min. The obtained supernatant was sterile filtrated with a 0.22 µm filter and was used for the assay. Standard preparation and assay procedure were done according to manufacturer’s instruction for colorimetric assays.

**Statistical information**

All infection experiments were performed a minimum of three times. Statistical parameters including dispersion and precision measures (mean ± SEM) and statistical significance are reported in the figures and figure legends. Data is judged to be statistically significant when p < 0.05 by two-tailed Student’s t-test. In figures, asterisks denote statistical significance as calculated by unpaired Student’s t-test with two-tailed analysis (^∗^, p < 0.05, ^∗∗^, p < 0.01, ^∗∗∗^, p < 0.001). Statistical analysis was performed in GraphPad Prism

**References**

1- Pfaffl, M.W. (2001) A new mathematical model for relative quantification in real-time RT-PCR. Nucleic acids research 29: e45.

2- Weber M & Weber F (2014) Monitoring activation of the antiviral pattern recognition receptors RIG-I and PKR by limited protease digestion and native PAGE. J Vis Exp (89):e51415.
