# Supplementary material for: The secRNome of Listeria monocytogenes Harbors Small Noncoding RNAs That Are Potent Inducers of Beta Interferon
Source: mBio. 2019 Oct 8;10(5):e01223-19. doi: 10.1128/mBio.01223-19 (PMC6786865; doi:10.1128/mBio.01223-19)
Supplement: TABLE S2 [file mBio.01223-19-st002.pdf]

Table S2. Differentially expressed mRNAs in sec-RNA, MVs-RNA and cytosolic RNA in the overexpression strain (Lm-rli32) and deletion mutant (Lm-Δrli32) as compared to the wild type (Lm)

A. sec-RNA (mRNA)

Lmo number	Product	Function
lmo0204	actin-assembly inducing protein precursor	Cell motility
lmo0205	phospholipase C	Defense/virulence mechanisms
lmo0375	hypothetical protein - FIG00774374: hypothetical protein	Not in COGs
lmo0422	lineage-specific thermal regulator protein	Transcription
lmo0423	RNA polymerase factor sigma C	Transcription
lmo0472	hypothetical protein	Not in COGs
lmo0475	hypothetical protein	Not in COGs
lmo0496	hypothetical protein - FIG00774392: hypothetical protein	Function unknown
lmo0540	hypothetical protein - penicillin-binding protein, putative	Defense/virulence mechanisms
lmo0615	hypothetical protein	Not in COGs
lmo0748	hypothetical protein - hypothetical protein	Not in COGs
lmo0751	hypothetical protein - FIG00775008: hypothetical protein	Not in COGs
lmo0815	hypothetical protein - Transcriptional regulator, MarR family	Transcription
lmo0866a	#NV	#NV
lmo1099	hypothetical protein - FIG00515940: hypothetical protein	Transcription; Signal transduction mechanisms;
lmo1216	hypothetical protein - N-acetylmuramoyl-L-alanine amidase, family 4	Cell motility; Intracellular trafficking and secretion;
lmo1776	hypothetical protein - FIG00774849: hypothetical protein	Function unknown
lmo1786	internalin C	Defense/virulence mechanisms
lmo2150	hypothetical protein - FIG00774336: hypothetical protein	Not in COGs
lmo2180	hypothetical protein - FIG00774300: hypothetical protein	Not in COGs
lmo2197	hypothetical protein - FIG00774927: hypothetical protein	Energy production and conversion
lmo2278	L-alanoyl-D-glutamate peptidase	Not in COGs
lmo2279	holin	Not in COGs
lmo2280	protein gp23	Not in COGs
lmo2281	protein gp22	Not in COGs

lmo2282	protein gp21	Not in COGs
lmo2283	protein gp20	Not in COGs
lmo2284	protein gp19	Not in COGs
lmo2285	protein gp18	Translation
lmo2286	protein gp17	Not in COGs
lmo2287	putative tape-measure	Not in COGs
lmo2292	protein gp11	Not in COGs
lmo2293	protein gp10	Not in COGs
lmo2295	protein gp8	Not in COGs
lmo2296	hypothetical protein - Phage capsid protein	Not in COGs
lmo2297	putative scaffolding protein	Cell motility; Signal transduction mechanisms;
lmo2564	hypothetical protein - 4-oxalocrotonate tautomerase (EC 5.3.2.-) Xylose transport system permease protein xylH	General function prediction only
lmo2707	hypothetical protein - FIG00774270: hypothetical protein	Not in COGs
lmo2711	hypothetical protein	Not in COGs

B. MVs-RNA (mRNA)

lmo0149	hypothetical protein	Not in COGs
lmo0321	hypothetical protein - membrane protein	Not in COGs
lmo0611	azoreductase	Lipid transport and metabolism
lmo0636	hypothetical protein - Rrf2 family transcriptional regulator	Transcription
lmo0637	hypothetical protein - 2-heptaprenyl-1,4-naphthoquinone methyltransferase (EC 2.1.1.-)	Secondary metabolites biosynthesis, transport and catabolism; General function prediction only;
lmo0642	hypothetical protein - membrane protein	Not in COGs
lmo0656	hypothetical protein - FIG00774201: hypothetical protein	Function unknown
lmo0787	hypothetical protein - D-serine/D-alanine/glycine transporter	Amino acid transport and metabolism
lmo0831	hypothetical protein - FIG00774061: hypothetical protein	General function prediction only
lmo0837	Pseudo-gene or RefSeq provisional	Not in COGs
lmo0998	hypothetical protein	General function prediction only
lmo1210	hypothetical protein - membrane protein	Function unknown
lmo1211	hypothetical protein - hypothetical protein	Function unknown

lmo1247	hypothetical protein - FIG00774866: hypothetical protein	Not in COGs
lmo1252	hypothetical protein - membrane protein	Function unknown
lmo1295	hypothetical protein - RNA-binding protein Hfq	Transcription; Translation;
lmo1300	hypothetical protein	Inorganic ion transport and metabolism
lmo1351	hypothetical protein - Rhodanese-like domain protein	Inorganic ion transport and metabolism
lmo1364	hypothetical protein - cold-shock protein	Transcription
lmo1385	hypothetical protein - hypothetical protein	Function unknown
lmo1388	CD4+ T cell-stimulating antigen, lipoprotein	General function prediction only
lmo1390	hypothetical protein - Unspecified monosaccharide ABC transport system, permease component Ia (FIG025991) / Unspecified monosaccharide ABC transport system, permease component Ib (FIG143636)	General function prediction only
lmo1391	hypothetical protein - Unspecified monosaccharide ABC transport system, permease component 2	General function prediction only
lmo1401	hypothetical protein - FIG006542: Phosphoesterase	Function unknown
lmo1416	hypothetical protein - FIG00774487: hypothetical protein	Defense/virulence mechanisms
lmo1417	hypothetical protein - membrane protein, putative	General function prediction only
lmo1445	transcriptional regulator ZurR (ferric uptake regulation)	Inorganic ion transport and metabolism
lmo1468	hypothetical protein - Transamidase GatB domain protein	Function unknown
lmo1476	coproporphyrinogen III oxidase	Coenzyme transport and metabolism
lmo1494	hypothetical protein - 5'-methylthioadenosine nucleosidase (EC 3.2.2.16) / S-adenosylhomocysteine nucleosidase (EC 3.2.2.9)	Nucleotide transport and metabolism
lmo1500	hypothetical protein - DedA family protein	Function unknown
lmo1521	hypothetical protein - N-acetylmuramoyl-L-alanine amidase (EC 3.5.1.28)	Signal transduction mechanisms; Cell wall/membrane biogenesis;
lmo1528	hypothetical protein - FIG00774068: hypothetical protein	Not in COGs
lmo1529	hypothetical protein - Preprotein translocase subunit YajC (TC 3.A.5.1.1)	Intracellular trafficking and secretion
lmo1536	prephenate dehydratase	Amino acid transport and metabolism
lmo1617	hypothetical protein	Carbohydrate transport and metabolism; Amino acid transport and metabolism; Inorganic ion transport and metabolism; General function prediction only;
lmo1623	hypothetical protein - FIG00775051: hypothetical protein	Lipid transport and metabolism
lmo1634	bifunctional acetaldehyde-CoA/alcohol dehydrogenase	Energy production and conversion

lmo1670	hypothetical protein - Protein YidD	Function unknown
lmo2057	protoheme IX farnesyltransferase	Posttranslational modification, protein turnover, chaperones
lmo2076	hypothetical protein	General function prediction only
lmo2078	hypothetical protein - ATPase YjeE, predicted to have essential role in cell wall biosynthesis	General function prediction only
lmo2088	hypothetical protein - Transcriptional regulator, TetR family	Transcription
lmo2127	hypothetical protein - CAAX amino terminal protease family protein	General function prediction only
lmo2145	hypothetical protein - Predicted nicotinate-regulated transporter BH3254	General function prediction only
lmo2158	hypothetical protein - FIG00774237: hypothetical protein	Function unknown
lmo2174	hypothetical protein - GGDEF domain protein	Signal transduction mechanisms
lmo2210	hypothetical protein - FIG00774091: hypothetical protein	Not in COGs
lmo2279	holin	Not in COGs
lmo2280	protein gp23	Not in COGs
lmo2281	protein gp22	Not in COGs
lmo2282	protein gp21	Not in COGs
lmo2288	protein gp15	Not in COGs
lmo2297	putative scaffolding protein	Cell motility; Signal transduction mechanisms;
lmo2305	hypothetical protein - Hypothetical protein, Lmo2305 homolog [Bacteriophage A118]	Not in COGs
lmo2307	hypothetical protein	Not in COGs
lmo2574	hypothetical protein - FIG00774193: hypothetical protein	Not in COGs
lmo2575	hypothetical protein - Cobalt-zinc-cadmium resistance protein CzcD	Inorganic ion transport and metabolism
lmo2680	potassium-transporting atpase c chain	Inorganic ion transport and metabolism
lmo2690	hypothetical protein - Transcriptional regulator, TetR family	Transcription
lmo2844	hypothetical protein - spermidine N1-acetyltransferase	Translation
**C. cytosolic-RNA (mRNA)**		
lmo0019	hypothetical protein - FIG00774048: hypothetical protein	Function unknown
lmo0021	hypothetical protein - PTS system, IIA component	Carbohydrate transport and metabolism
lmo0025	hypothetical protein - FIG00774489: hypothetical protein	General function prediction only
lmo0026	hypothetical protein	Inorganic ion transport and metabolism
lmo0034	hypothetical protein - PTS system, cellobiose-specific IIC component (EC 2.7.1.69)	Carbohydrate transport and metabolism

lmo0036	putrescine carbamoyltransferase	Amino acid transport and metabolism
lmo0037	hypothetical protein - Agmatine/putrescine antiporter, associated with agmatine catabolism	Amino acid transport and metabolism
lmo0043	arginine deiminase	Amino acid transport and metabolism
lmo0049	hypothetical protein - accessory gene regulator protein D, putative	Not in COGs
lmo0081	hypothetical protein - hypothetical protein	Not in COGs
lmo0082	hypothetical protein	Not in COGs
lmo0094	hypothetical protein - FIG00774358: hypothetical protein	Not in COGs
lmo0098	hypothetical protein - PTS system, mannose-specific IID component (EC 2.7.1.69)	Carbohydrate transport and metabolism
lmo0115	hypothetical protein - Listeria protein LmaD, associated with virulence	Not in COGs
lmo0116	hypothetical protein - LmaC, associated with virulence in Listeria	Not in COGs
lmo0117	antigen B	Not in COGs
lmo0118	antigen A	Function unknown
lmo0119	hypothetical protein - FIG00774706: hypothetical protein	Not in COGs
lmo0120	hypothetical protein - FIG00774110: hypothetical protein	Not in COGs
lmo0128	hypothetical protein - N-acetylmuramoyl-L-alanine amidase (EC 3.5.1.28)	General function prediction only
lmo0129	hypothetical protein - N-acetylmuramoyl-L-alanine amidase (EC 3.5.1.28)	Cell wall/membrane biogenesis
lmo0133	hypothetical protein - FIG00774781: hypothetical protein	Function unknown
lmo0135	hypothetical protein - Oligopeptide ABC transporter, periplasmic oligopeptide-binding protein OppA (TC 3.A.1.5.1)	Amino acid transport and metabolism
lmo0136	hypothetical protein - Oligopeptide transport system permease protein OppB (TC 3.A.1.5.1)	Amino acid transport and metabolism; Inorganic ion transport and metabolism;
lmo0137	hypothetical protein	Amino acid transport and metabolism; Inorganic ion transport and metabolism;
lmo0146	hypothetical protein - FIG00774118: hypothetical protein	Not in COGs
lmo0152	hypothetical protein - Oligopeptide ABC transporter, periplasmic oligopeptide-binding protein OppA (TC 3.A.1.5.1)	Amino acid transport and metabolism
lmo0153	hypothetical protein - Zinc ABC transporter, periplasmic-binding protein ZnuA	Inorganic ion transport and metabolism
lmo0154	hypothetical protein - Zinc ABC transporter, ATP-binding protein ZnuC	Inorganic ion transport and metabolism
lmo0155	hypothetical protein	Inorganic ion transport and metabolism
lmo0169	hypothetical protein - glucose uptake protein	Carbohydrate transport and metabolism
lmo0170	hypothetical protein	Function unknown
lmo0172	Pseudo-gene or RefSeq provisional	Not in COGs

**lmo0178**	**hypothetical protein - Putative ROK-family transcriptional regulator**	**Transcription; Carbohydrate transport and metabolism;**
**lmo0179**	**hypothetical protein - N-Acetyl-D-glucosamine ABC transport system, permease protein 1**	**Carbohydrate transport and metabolism**
**lmo0181**	**hypothetical protein - N-Acetyl-D-glucosamine ABC transport system, sugar-binding protein**	**Carbohydrate transport and metabolism**
**lmo0186**	**hypothetical protein - Cell wall-binding protein**	**Function unknown**
**lmo01876**	**Pseudo-gene or RefSeq provisional**	**Not in COGs**
**lmo0189**	**hypothetical protein - Veg protein**	**Function unknown**
**lmo0200**	**listeriolysin positive regulatory protein**	**Transcription**
**lmo0202**	**listeriolysin O precursor**	**Defense/virulence mechanisms**
**lmo0203**	**Zinc metalloproteinase precursor**	**Amino acid transport and metabolism**
**lmo0204**	**actin-assembly inducing protein precursor**	**Cell motility**
**lmo0205**	**phospholipase C**	**Defense/virulence mechanisms**
**lmo0206**	**hypothetical protein**	**Not in COGs**
**lmo0207**	**hypothetical protein - hypothetical lipoprotein**	**Function unknown**
**lmo0221**	**pantothenate kinase**	**Transcription**
**lmo0223**	**hypothetical protein - Cysteine synthase (EC 2.5.1.47)**	**Amino acid transport and metabolism**
**lmo02333**	**Pseudo-gene or RefSeq provisional**	**Not in COGs**
**lmo0254**	**hypothetical protein - FIG00774737: hypothetical protein**	**Not in COGs**
**lmo0255**	**hypothetical protein - hypothetical lipoprotein**	**Function unknown**
**lmo0263**	**internalin H**	**Cell wall/membrane biogenesis**
**lmo0264**	**internalin E**	**Cell wall/membrane biogenesis**
**lmo0274**	**hypothetical protein - FIG00774862: hypothetical protein**	**Signal transduction mechanisms**
**lmo0293**	**rRNA large subunit methyltransferase**	**Function unknown**
**lmo0320**	**hypothetical protein - Putative peptidoglycan bound protein (LPXTG motif) Lmo0320 homolog**	**Cell wall/membrane biogenesis**
**lmo0321**	**hypothetical protein - membrane protein**	**Not in COGs**
**lmo0322**	**hypothetical protein - FIG00774312: hypothetical protein**	**Not in COGs**
**lmo0325**	**hypothetical protein - Transcriptional regulator, MutR family**	**Transcription**
**lmo0331**	**hypothetical protein - Internalin-like protein (LPXTG motif) Lmo0331 homolog**	**Function unknown**
**lmo0332**	**hypothetical protein - FIG00774436: hypothetical protein**	**Not in COGs**

lmo0338	hypothetical protein - FIG00774512: hypothetical protein	Not in COGs
lmo0361	hypothetical protein	Intracellular trafficking and secretion
lmo0362	hypothetical protein - Twin-arginine translocation protein TatA	Intracellular trafficking and secretion
lmo0365	hypothetical protein - Ferrous iron transport permease EfeU	Inorganic ion transport and metabolism
lmo0366	hypothetical protein - Ferrous iron transport periplasmic protein EfeO, contains peptidase-M75 domain and (frequently) cupredoxin-like domain	Inorganic ion transport and metabolism
lmo0367	hypothetical protein - Ferrous iron transport peroxidase EfeB	Inorganic ion transport and metabolism
lmo0368	hypothetical protein - Putative Nudix hydrolase YfcD (EC 3.6.-.-)	Replication, recombination and repair; General function prediction only;
lmo0374	hypothetical protein - PTS system, cellobiose-specific IIB component (EC 2.7.1.69)	Carbohydrate transport and metabolism
lmo0375	hypothetical protein - FIG00774374: hypothetical protein	Not in COGs
lmo0382	hypothetical protein - Transcriptional repressor of the myo-inositol catabolic operon DeoR family	Transcription; Carbohydrate transport and metabolism;
lmo0383	hypothetical protein - Methylmalonate-semialdehyde dehydrogenase [inositol] (EC 1.2.1.27)	Energy production and conversion
lmo0384	hypothetical protein - 5-deoxy-glucuronate isomerase (EC 5.3.1.-)	Carbohydrate transport and metabolism
lmo0385	hypothetical protein - 5-keto-2-deoxygluconokinase (EC 2.7.1.92)	Carbohydrate transport and metabolism
lmo0386	hypothetical protein	Amino acid transport and metabolism
lmo0392	hypothetical protein - DUF1432 domain-containing protein	Function unknown
lmo0393	hypothetical protein - FIG00774100: hypothetical protein	Not in COGs
lmo0403	hypothetical protein - FIG00774247: hypothetical protein	Not in COGs
lmo0404	hypothetical protein - FIG00774624: hypothetical protein	Not in COGs
lmo0405	hypothetical protein - Probable low-affinity inorganic phosphate transporter	Inorganic ion transport and metabolism
lmo0412	hypothetical protein	Not in COGs
lmo0421	hypothetical protein - Cell division protein FtsW	Cell cycle control, mitosis and meiosis
lmo0422	lineage-specific thermal regulator protein	Transcription
lmo0423	RNA polymerase factor sigma C	Transcription
lmo0435	peptidoglycan binding protein	Not in COGs
lmo0439	hypothetical protein - Siderophore/Surfactin synthetase related protein	General function prediction only
lmo0445	hypothetical protein - FIG00775532: hypothetical protein	Transcription
lmo0465	hypothetical protein	Not in COGs
lmo0469	hypothetical protein - FIG00776455: hypothetical protein	Not in COGs

lmo0470	hypothetical protein - Adenine-specific methyltransferase (EC 2.1.1.72)	Replication, recombination and repair
lmo0471	hypothetical protein - hypothetical protein	Not in COGs
lmo0475	hypothetical protein	Not in COGs
lmo0484	heme-degrading monooxygenase IsdG	General function prediction only
lmo0485	hypothetical protein - Putative nitroreductase family protein SACOL0874	Energy production and conversion
lmo0492	hypothetical protein - transcriptional regulator, LysR family	Transcription
lmo0496	hypothetical protein - FIG00774392: hypothetical protein	Function unknown
lmo0519	hypothetical protein - drug resistance transporter, EmrB/QacA family	Carbohydrate transport and metabolism; Amino acid transport and metabolism; Inorganic ion transport and metabolism; General function prediction only;
lmo0520	hypothetical protein	Transcription; Carbohydrate transport and metabolism;
lmo0539	tagatose 1,6-diphosphate aldolase	Carbohydrate transport and metabolism
lmo0540	hypothetical protein - penicillin-binding protein, putative	Defense/virulence mechanisms
lmo0541	hypothetical protein - ABC transporter, substrate-binding protein	Inorganic ion transport and metabolism
lmo0546	hypothetical protein - Sorbitol-6-phosphate 2-dehydrogenase (EC 1.1.1.140)	Amino acid transport and metabolism
lmo0556	hypothetical protein - Phosphoglycerate mutase family, Lmo0556 homolog	Carbohydrate transport and metabolism
lmo0589	hypothetical protein - FIG00774318: hypothetical protein	Function unknown
lmo0590	hypothetical protein	General function prediction only; Function unknown;
lmo0591	hypothetical protein - FIG00775146: hypothetical protein	Function unknown
lmo0593	hypothetical protein - formate/nitrite transporter family protein	Inorganic ion transport and metabolism
lmo0596	hypothetical protein - FIG00774040: hypothetical protein	Function unknown
lmo0597	hypothetical protein - cyclic nucleotide-binding protein	Signal transduction mechanisms
lmo0599	hypothetical protein - Transcriptional regulator, PadR family	Transcription
lmo0600	hypothetical protein - FIG00774777: hypothetical protein	Function unknown
lmo0602	hypothetical protein - acetyltransferase, GNAT family	Transcription; General function prediction only;
lmo0610	hypothetical protein - Internalin-like protein (LPXTG motif) Lmo0610 homolog	General function prediction only; Function unknown;
lmo0622	hypothetical protein - FIG00774605: hypothetical protein	Not in COGs
lmo0628	hypothetical protein	Not in COGs

lmo0636	hypothetical protein - Rrf2 family transcriptional regulator	Transcription
lmo0637	hypothetical protein - 2-heptaprenyl-1,4-naphthoquinone methyltransferase (EC 2.1.1.-)	Secondary metabolites biosynthesis, transport and catabolism; General function prediction only;
lmo0641	hypothetical protein - Lead, cadmium, zinc and mercury transporting ATPase (EC 3.6.3.3) (EC 3.6.3.5) Copper-translocating P-type ATPase (EC 3.6.3.4)	Inorganic ion transport and metabolism
lmo0642	hypothetical protein - membrane protein	Not in COGs
lmo0646	hypothetical protein - Glyoxalase family protein	General function prediction only
lmo0647	hypothetical protein - FIG00774323: hypothetical protein	Not in COGs
lmo0648	hypothetical protein - Magnesium and cobalt transport protein CorA	Inorganic ion transport and metabolism
lmo0654	hypothetical protein - FIG00774719: hypothetical protein	Not in COGs
lmo0655	hypothetical protein - Serine/threonine protein phosphatase (EC 3.1.3.16)	Signal transduction mechanisms
lmo0659	hypothetical protein - Transcriptional regulator, MutR family	Transcription
lmo0661	hypothetical protein - carboxymuconolactone decarboxylase family protein	Function unknown
lmo0672	hypothetical protein - hypothetical protein	Function unknown
lmo0673	hypothetical protein - FIG00774179: hypothetical protein	Not in COGs
lmo0692	two-component sensor histidine kinase CheA	Cell motility; Signal transduction mechanisms;
lmo0693	flagellar motor switch protein	Cell motility; Intracellular trafficking and secretion;
lmo0698	flagellar motor switch protein	Cell motility; Intracellular trafficking and secretion;
lmo0704	hypothetical protein - FIG00774686: hypothetical protein	Not in COGs
lmo0705	flagellar hook-associated protein FlgK	Cell motility
lmo0706	flagellar hook-associated protein FlgL	Cell motility
lmo0707	flagellar capping protein	Cell motility
lmo0709	hypothetical protein - FIG00774899: hypothetical protein	Not in COGs
lmo0710	flagellar basal body rod protein FlgB	Cell motility
lmo0711	flagellar basal body rod protein FlgC	Cell motility
lmo0712	flagellar hook-basal body protein FliE	Cell motility; Intracellular trafficking and secretion;
lmo0713	flagellar MS-ring protein	Cell motility; Intracellular trafficking and secretion;
lmo0714	flagellar motor switch protein G	Cell motility
lmo0726	hypothetical protein	Not in COGs
lmo0727	glucosamine--fructose-6-phosphate aminotransferase	Cell wall/membrane biogenesis

lmo0746	hypothetical protein - FIG00774480: hypothetical protein	Not in COGs
lmo0748	hypothetical protein - hypothetical protein	Not in COGs
lmo0750	hypothetical protein - FIG00774837: hypothetical protein	Not in COGs
lmo0751	hypothetical protein - FIG00775008: hypothetical protein	Not in COGs
lmo0776	hypothetical protein - Fructokinase (EC 2.7.1.4)	Transcription; Carbohydrate transport and metabolism;
lmo0778	hypothetical protein	Not in COGs
lmo0780	hypothetical protein - FIG00775457: hypothetical protein	Not in COGs
lmo0782	hypothetical protein - PTS system, mannose-specific IIC component (EC 2.7.1.69) / PTS system, fructose-specific IIC component (EC 2.7.1.69)	Carbohydrate transport and metabolism
lmo0783	hypothetical protein - PTS system, mannose-specific IIB component (EC 2.7.1.69)	Carbohydrate transport and metabolism
lmo0784	hypothetical protein - PTS system, mannose-specific IIB component (EC 2.7.1.69) / PTS system, mannose-specific IIA component (EC 2.7.1.69)	Carbohydrate transport and metabolism
lmo0788	hypothetical protein - Activator of (R)-2-hydroxyglutaryl-CoA dehydratase	Lipid transport and metabolism; Function unknown;
lmo0794	hypothetical protein - Rrf2-linked NADH-flavin reductase	General function prediction only
lmo0800	hypothetical protein - FIG00774434: hypothetical protein	Function unknown
lmo0813	hypothetical protein - Fructokinase (EC 2.7.1.4)	Transcription; Carbohydrate transport and metabolism;
lmo0815	hypothetical protein - Transcriptional regulator, MarR family	Transcription
lmo0816	hypothetical protein - protease synthase and sporulation negative regulatory protein pai 1	Transcription; General function prediction only;
lmo0823	hypothetical protein - oxidoreductase of aldo/keto reductase family, subgroup 1	General function prediction only
lmo0833	hypothetical protein - transcriptional activator	Transcription
lmo0838	sugar phosphate antiporter	Carbohydrate transport and metabolism
lmo0839	hypothetical protein - Multidrug-efflux transporter, major facilitator superfamily (MFS) (TC 2.A.1) Efflux pump Lde	Carbohydrate transport and metabolism; Amino acid transport and metabolism; Inorganic ion transport and metabolism; General function prediction only;
lmo0845	hypothetical protein - Methionine synthase II (cobalamin-independent)	Amino acid transport and metabolism
lmo0850	hypothetical protein - FIG00774105: hypothetical protein	Not in COGs
lmo0859	hypothetical protein - Multiple sugar ABC transporter, substrate-binding protein	Carbohydrate transport and metabolism
lmo0866a	#NV	#NV

lmo0867	hypothetical protein - Substrate-specific component QueT (COG4708) of predicted queuosine-regulated ECF transporter	Function unknown
lmo0869	hypothetical protein - 2-amino-3-carboxymuconate-6-semialdehyde decarboxylase (EC 4.1.1.45)	General function prediction only
lmo0871	hypothetical protein - Transcriptional regulator, HxlR family	Transcription
lmo0880	hypothetical protein - Putative peptidoglycan bound protein (LPXTG motif) Lmo0880 homolog	Cell wall/membrane biogenesis
lmo0881	hypothetical protein - Lmo0881 homolog, only in Listeria	Not in COGs
lmo0903	hypothetical protein - OsmC/Ohr family protein	Posttranslational modification, protein turnover, chaperones
lmo0913	hypothetical protein - Succinate-semialdehyde dehydrogenase [NAD] (EC 1.2.1.24) Succinate-semialdehyde dehydrogenase [NADP+] (EC 1.2.1.16)	Energy production and conversion
lmo0914	hypothetical protein - PTS system, cellobiose-specific IIB component (EC 2.7.1.69)	Carbohydrate transport and metabolism
lmo0915	hypothetical protein - PTS system, cellobiose-specific IIC component (EC 2.7.1.69)	Carbohydrate transport and metabolism
lmo0916	hypothetical protein - PTS system, cellobiose-specific IIA component (EC 2.7.1.69)	Carbohydrate transport and metabolism
lmo0917	hypothetical protein - 6-phospho-beta-glucosidase (EC 3.2.1.86)	Carbohydrate transport and metabolism
lmo0932	hypothetical protein - FIG00774802: hypothetical protein	Function unknown
lmo0937	hypothetical protein - hypothetical protein	Not in COGs
lmo0977	hypothetical protein - Putative acetyl esterase YjcH (EC 3.1.1.-)	Inorganic ion transport and metabolism
lmo0979	hypothetical protein - Methionine ABC transporter ATP-binding protein	Defense/virulence mechanisms
lmo0990	hypothetical protein - Multi antimicrobial extrusion protein (Na(+)/drug antiporter), MATE family of MDR efflux pumps	Defense/virulence mechanisms
lmo0994	hypothetical protein - FIG00774070: hypothetical protein	Not in COGs
lmo0995	hypothetical protein - membrane protein	Carbohydrate transport and metabolism
lmo1007	hypothetical protein - FIG00775035: hypothetical protein	Not in COGs
lmo1014	hypothetical protein - Glycine betaine ABC transport system, ATP-binding protein OpuAA (EC 3.6.3.32)	Amino acid transport and metabolism
lmo1028	hypothetical protein - Protein of unknown function DUF1447	Function unknown
lmo1033	hypothetical protein - Transketolase, C-terminal section (EC 2.2.1.1)	Carbohydrate transport and metabolism
lmo1035	hypothetical protein - PTS system, beta-glucoside-specific IIB component (EC 2.7.1.69) / PTS system, beta-glucoside-specific IIC component (EC 2.7.1.69) / PTS system, beta-glucoside-specific IIA component (EC 2.7.1.69)	Carbohydrate transport and metabolism
lmo1067	hypothetical protein - GTP-binding protein TypA/BipA	Signal transduction mechanisms
lmo1102	hypothetical protein - Cadmium efflux system accessory protein	Transcription

lmo1112	hypothetical protein - FIG00628088: hypothetical protein	Cell cycle control, mitosis and meiosis
lmo1113	hypothetical protein	Not in COGs
lmo1114	hypothetical protein - FIG00627241: hypothetical protein	Not in COGs
lmo1131	hypothetical protein - Transport ATP-binding protein CydC	Energy production and conversion; Posttranslational modification, protein turnover, chaperones;
lmo1132	hypothetical protein - Transport ATP-binding protein CydD	Defense/virulence mechanisms
lmo1137	hypothetical protein - FIG00774175: hypothetical protein	Function unknown
lmo1142	hypothetical protein	Energy production and conversion
lmo1143	hypothetical protein - Propanediol utilization polyhedral body protein PduT	Secondary metabolites biosynthesis, transport and catabolism; Energy production and conversion;
lmo1147	hypothetical protein - Adenosylcobinamide-phosphate guanylyltransferase (EC 2.7.7.62)	Coenzyme transport and metabolism
lmo1190	hypothetical protein - Substrate-specific component CblT of predicted B12-regulated ECF transporter for dimethylbenzimidazole	Not in COGs
lmo1211	hypothetical protein - hypothetical protein	Function unknown
lmo1216	hypothetical protein - N-acetylmuramoyl-L-alanine amidase, family 4	Cell motility; Intracellular trafficking and secretion;
lmo1227	uracil-DNA glycosylase	Replication, recombination and repair
lmo1245	hypothetical protein - FIG00774287: hypothetical protein	Not in COGs
lmo1249	hypothetical protein - FIG00774262: hypothetical protein	Not in COGs
lmo1250	hypothetical protein - transporter	Carbohydrate transport and metabolism; Amino acid transport and metabolism; Inorganic ion transport and metabolism; General function prediction only;
lmo1251	hypothetical protein - transcriptional regulator, Crp/Fnr family	Signal transduction mechanisms
lmo1252	hypothetical protein - membrane protein	Function unknown
lmo1256	hypothetical protein - FIG00774111: hypothetical protein	Replication, recombination and repair; General function prediction only;
lmo1257	hypothetical protein - FIG00774071: hypothetical protein	Not in COGs
lmo1261	hypothetical protein - membrane protein	Function unknown
lmo1266	hypothetical protein - FIG00774727: hypothetical protein	General function prediction only
lmo1295	hypothetical protein - RNA-binding protein Hfq	Transcription; Translation;
lmo1298	hypothetical protein	Transcription

lmo1299	hypothetical protein - Glutamine synthetase type I (EC 6.3.1.2)	Amino acid transport and metabolism
lmo1303	cell division suppressor protein YneA	Cell wall/membrane biogenesis
lmo1304	hypothetical protein - FIG00774411: hypothetical protein	Function unknown
lmo1306	hypothetical protein - FIG00774758: hypothetical protein	Function unknown
lmo1335	50S ribosomal protein L33	Translation
lmo1338	hypothetical protein - FIG00774150: hypothetical protein	Function unknown
lmo1340	hypothetical protein - lipoprotein	Not in COGs
lmo1344	hypothetical protein - Late competence protein ComGD, access of DNA to ComEA, FIG012777	Cell motility; Intracellular trafficking and secretion;
lmo1364	hypothetical protein - cold-shock protein	Transcription
lmo1368	DNA repair and genetic recombination	Replication, recombination and repair
lmo1369	hypothetical protein - Phosphate butyryltransferase (EC 2.3.1.19)	Energy production and conversion
lmo1404	DNA mismatch repair protein	Replication, recombination and repair
lmo1406	pyruvate formate-lyase	Energy production and conversion
lmo1407	pyruvate-formate lyase activating enzyme	Posttranslational modification, protein turnover, chaperones
lmo1421	hypothetical protein - Glycine betaine ABC transport system, ATP-binding protein OpuAA (EC 3.6.3.32)	Defense/virulence mechanisms
lmo1422	hypothetical protein - Glycine betaine ABC transport system, permease protein OpuAB / Glycine betaine ABC transport system, glycine betaine-binding protein OpuAC	Defense/virulence mechanisms
lmo1428	hypothetical protein - Osmotically activated L-carnitine/choline ABC transporter, ATP-binding protein OpuCA	Amino acid transport and metabolism
lmo1432	hypothetical protein - FIG00774303: hypothetical protein	Not in COGs
lmo1433	hypothetical protein - Glutathione reductase (EC 1.8.1.7)	Energy production and conversion
lmo1439	superoxide dismutase	Inorganic ion transport and metabolism
lmo1444	hypothetical protein - Foldase protein PrsA precursor (EC 5.2.1.8)	Posttranslational modification, protein turnover, chaperones
lmo1445	transcriptional regulator ZurR (ferric uptake regulation)	Inorganic ion transport and metabolism
lmo1446	metal (zinc) transport protein (ABC transporter, permease protein)	Inorganic ion transport and metabolism
lmo1464	hypothetical protein	Cell wall/membrane biogenesis
lmo1473	molecular chaperone DnaK	Posttranslational modification, protein turnover, chaperones

lmo1474	heat shock protein GrpE	Posttranslational modification, protein turnover, chaperones
lmo1475	heat-inducible transcription repressor	Transcription
lmo1516	hypothetical protein - Ammonium transporter	Inorganic ion transport and metabolism
lmo1517	hypothetical protein - Nitrogen regulatory protein P-II	Amino acid transport and metabolism
lmo1518	hypothetical protein - FIG00774153: hypothetical protein	Not in COGs
lmo1526	hypothetical protein - FIG00774752: hypothetical protein	Function unknown
lmo1529	hypothetical protein - Preprotein translocase subunit YajC (TC 3.A.5.1.1)	Intracellular trafficking and secretion
lmo1552	valyl-tRNA synthetase	Translation
lmo1575	hypothetical protein - FIG146085: 3'-to-5' oligoribonuclease A, Bacillus type	General function prediction only
lmo1576	hypothetical protein	Transcription
lmo1580	hypothetical protein - Universal stress protein family	Signal transduction mechanisms
lmo1601	hypothetical protein - general stress protein	General function prediction only
lmo1602	hypothetical protein - hypothetical protein	General function prediction only
lmo1618	hypothetical protein - Transcriptional regulator, MarR family	Transcription
lmo1623	hypothetical protein - FIG00775051: hypothetical protein	Lipid transport and metabolism
lmo1624	hypothetical protein - Membrane protein involved in the export of O-antigen, teichoic acid lipoteichoic acids	General function prediction only
lmo1625	hypothetical protein	General function prediction only
lmo1626	hypothetical protein - FIG00774419: hypothetical protein	Not in COGs
lmo1627	tryptophan synthase subunit alpha	Amino acid transport and metabolism
lmo1628	tryptophan synthase subunit beta	Amino acid transport and metabolism
lmo1629	N-(5'-phosphoribosyl)anthranilate isomerase	Amino acid transport and metabolism
lmo1630	indole-3-glycerol-phosphate synthase	Amino acid transport and metabolism
lmo1631	anthranilate phosphoribosyltransferase	Amino acid transport and metabolism
lmo1632	anthranilate synthase component II	Amino acid transport and metabolism; Coenzyme transport and metabolism;
lmo1633	anthranilate synthase component I	Amino acid transport and metabolism; Coenzyme transport and metabolism;
lmo1634	bifunctional acetaldehyde-CoA/alcohol dehydrogenase	Energy production and conversion
lmo1637	hypothetical protein - ABC transporter, permease protein	General function prediction only
lmo1665	hypothetical protein - FIG00774797: hypothetical protein	Not in COGs

lmo1671	hypothetical protein - Lmo1671 protein	Inorganic ion transport and metabolism
lmo1678	bifunctional homocysteine S-methyltransferase/5,10-methylenetetrahydrofolate reductase protein	Amino acid transport and metabolism
lmo1679	cystathionine beta-lyase	Amino acid transport and metabolism
lmo1680	hypothetical protein - Cystathionine gamma-synthase (EC 2.5.1.48)	Amino acid transport and metabolism
lmo1681	5-methyltetrahydropteroyltriglutamate--homocysteine S-methyltransferase	Amino acid transport and metabolism
lmo1694	hypothetical protein - conserved hypothetical protein	General function prediction only
lmo1699	hypothetical protein - methyl-accepting chemotaxis protein	Cell motility; Signal transduction mechanisms;
lmo1700	hypothetical protein - FIG00774912: hypothetical protein	Not in COGs
lmo1705	hypothetical protein - Deoxyadenosine kinase (EC 2.7.1.76) / Deoxyguanosine kinase (EC 2.7.1.113)	Nucleotide transport and metabolism
lmo1707	hypothetical protein	Function unknown
lmo1725	hypothetical protein - Transcriptional regulator, GntR family	Transcription
lmo1733	glutamate synthase subunit beta	Amino acid transport and metabolism; General function prediction only;
lmo1734	hypothetical protein - Glutamate synthase [NADPH] large chain (EC 1.4.1.13)	Amino acid transport and metabolism
lmo1736	hypothetical protein - Acetyltransferase	General function prediction only
lmo1737	hypothetical protein	Energy production and conversion
lmo1738	hypothetical protein - Amino acid ABC transporter, amino acid-binding protein	Amino acid transport and metabolism; Signal transduction mechanisms;
lmo1739	hypothetical protein - amino acid ABC transporter, ATP-binding protein	Amino acid transport and metabolism
lmo1740	hypothetical protein - ABC transporter membrane-spanning permease - glutamine transport	Amino acid transport and metabolism
lmo1752	hypothetical protein - FIG00774698: hypothetical protein	Not in COGs
lmo1764	phosphoribosylamine--glycine ligase	Nucleotide transport and metabolism
lmo1765	bifunctional phosphoribosylaminoimidazolecarboxamide formyltransferase/IMP cyclohydrolase	Nucleotide transport and metabolism
lmo1769	phosphoribosylformylglycinamidine synthase II	Nucleotide transport and metabolism
lmo1770	phosphoribosylformylglycinamidine synthase I	Nucleotide transport and metabolism
lmo1772	phosphoribosylaminoimidazole-succinocarboxamide synthase	Nucleotide transport and metabolism
lmo1773	adenylosuccinate lyase	Nucleotide transport and metabolism
lmo1774	phosphoribosylaminoimidazole carboxylase ATPase subunit	Nucleotide transport and metabolism
lmo1775	phosphoribosylaminoimidazole carboxylase catalytic subunit	Nucleotide transport and metabolism

lmo1776	hypothetical protein - FIG00774849: hypothetical protein	Function unknown
lmo1786	internalin C	Defense/virulence mechanisms
lmo1789	hypothetical protein - flavodoxin-like fold domain protein	General function prediction only
lmo1834	hypothetical protein - Dihydroorotate dehydrogenase electron transfer subunit (EC 1.3.3.1)	Coenzyme transport and metabolism; Energy production and conversion;
lmo1835	carbamoyl phosphate synthase large subunit	Amino acid transport and metabolism; Nucleotide transport and metabolism;
lmo1836	carbamoyl phosphate synthase small subunit	Amino acid transport and metabolism; Nucleotide transport and metabolism;
lmo1837	dihydroorotase	Nucleotide transport and metabolism
lmo1838	aspartate carbamoyltransferase catalytic subunit	Nucleotide transport and metabolism
lmo1839	hypothetical protein - Uracil permease	Nucleotide transport and metabolism
lmo1840	bifunctional pyrimidine regulatory protein PyrR uracil phosphoribosyltransferase	Nucleotide transport and metabolism
lmo1847	hypothetical protein - Manganese ABC transporter, periplasmic-binding protein SitA	Cell wall/membrane biogenesis
lmo1848	hypothetical protein - Manganese ABC transporter, inner membrane permease protein SitD	Inorganic ion transport and metabolism
lmo1849	hypothetical protein - Manganese ABC transporter, ATP-binding protein SitB	Inorganic ion transport and metabolism
lmo1868	hypothetical protein - Lactoylglutathione lyase	Amino acid transport and metabolism
lmo1869	hypothetical protein - FIG00774701: hypothetical protein	Function unknown
lmo1877	hypothetical protein - Formate--tetrahydrofolate ligase (EC 6.3.4.3)	Nucleotide transport and metabolism
lmo1882	30S ribosomal protein S14	Translation
lmo1890	hypothetical protein - hyothetical protein	Not in COGs
lmo1912	hypothetical protein - GGDEF domain protein	Signal transduction mechanisms
lmo1944	hypothetical protein - Ferredoxin	Energy production and conversion
lmo1956	hypothetical protein - Ferric uptake regulation protein FUR	Inorganic ion transport and metabolism
lmo1957	hypothetical protein - ABC-type Fe3+-siderophore transport system, permease 2 component	Inorganic ion transport and metabolism
lmo1958	hypothetical protein - ABC-type Fe3+-siderophore transport system, permease component	Inorganic ion transport and metabolism
lmo1959	hypothetical protein - Ferrichrome-binding periplasmic protein precursor (TC 3.A.1.14.3)	Inorganic ion transport and metabolism
lmo1960	hypothetical protein - ABC-type Fe3+-siderophore transport system, ATPase component	Inorganic ion transport and metabolism; Coenzyme transport and metabolism;
lmo1961	hypothetical protein - Thioredoxin reductase (EC 1.8.1.9)	Posttranslational modification, protein turnover, chaperones
lmo1963	hypothetical protein	General function prediction only

lmo1964	hypothetical protein - ABC transporter, ATP-binding protein	Defense/virulence mechanisms
lmo1983	dihydroxy-acid dehydratase	Amino acid transport and metabolism; Carbohydrate transport and metabolism;
lmo1984	hypothetical protein - Acetolactate synthase large subunit (EC 2.2.1.6)	Amino acid transport and metabolism; Coenzyme transport and metabolism;
lmo1985	acetolactate synthase 3 regulatory subunit	Amino acid transport and metabolism
lmo1986	ketol-acid reductoisomerase	Amino acid transport and metabolism; Coenzyme transport and metabolism;
lmo1987	2-isopropylmalate synthase	Amino acid transport and metabolism
lmo1988	3-isopropylmalate dehydrogenase	Energy production and conversion; Amino acid transport and metabolism;
lmo1989	isopropylmalate isomerase large subunit	Amino acid transport and metabolism
lmo1990	isopropylmalate isomerase small subunit	Amino acid transport and metabolism
lmo1991	threonine dehydratase	Amino acid transport and metabolism
lmo2002	hypothetical protein - PTS system, mannose-specific IIB component (EC 2.7.1.69)	Carbohydrate transport and metabolism
lmo2004	hypothetical protein - Transcriptional regulator, GntR family	Transcription
lmo2006	acetolactate synthase	Amino acid transport and metabolism; Coenzyme transport and metabolism;
lmo2022	hypothetical protein - Cysteine desulfurase (EC 2.8.1.7)	Amino acid transport and metabolism
lmo2023	L-aspartate oxidase	Coenzyme transport and metabolism
lmo2024	nicotinate-nucleotide pyrophosphorylase	Coenzyme transport and metabolism
lmo2025	quinolinate synthetase	Coenzyme transport and metabolism
lmo2068	chaperonin GroEL	Posttranslational modification, protein turnover, chaperones
lmo2069	co-chaperonin GroES	Posttranslational modification, protein turnover, chaperones
lmo2070	hypothetical protein - CAAX amino terminal protease family protein	General function prediction only
lmo2071	hypothetical protein - FIG00774125: hypothetical protein	Not in COGs
lmo2081	camphor resistance protein CrcB	Cell cycle control, mitosis and meiosis
lmo2104	hypothetical protein - Hypothetical protein FIG016644	Inorganic ion transport and metabolism
lmo2104a	#NV	#NV
lmo2105	hypothetical protein - Ferrous iron transport protein B	Inorganic ion transport and metabolism
lmo2107	hypothetical protein - Transcriptional regulator, DeoR family	Transcription; Carbohydrate transport and metabolism;

lmo2108	hypothetical protein - N-acetylglucosamine-6-phosphate deacetylase (EC 3.5.1.25)	Carbohydrate transport and metabolism
lmo2109	hypothetical protein - hydrolase, alpha/beta fold family	General function prediction only
lmo2130	hypothetical protein - Amino acid permease	Amino acid transport and metabolism
lmo2131	hypothetical protein - FIG00774656: hypothetical protein	Signal transduction mechanisms
lmo2132	hypothetical protein - FIG00774113: hypothetical protein	Signal transduction mechanisms
lmo2145	hypothetical protein - Predicted nicotinate-regulated transporter BH3254	General function prediction only
lmo2151	hypothetical protein - FIG00774036: hypothetical protein	General function prediction only
lmo2152	hypothetical protein - thioredoxin, putative	Posttranslational modification, protein turnover, chaperones; Energy production and conversion;
lmo2154	ribonucleotide-diphosphate reductase subunit beta	Nucleotide transport and metabolism
lmo2156	hypothetical protein - FIG00774317: hypothetical protein	Function unknown
lmo2158	hypothetical protein - FIG00774237: hypothetical protein	Function unknown
lmo2161	hypothetical protein - FIG00774369: hypothetical protein	Carbohydrate transport and metabolism
lmo2172	hypothetical protein - Acetyl-CoA:acetoacetyl-CoA transferase, alpha subunit (EC 2.8.3.8)	Lipid transport and metabolism
lmo2177	hypothetical protein - FIG01225675: hypothetical protein	Not in COGs
lmo2180	hypothetical protein - FIG00774300: hypothetical protein	Not in COGs
lmo2181	hypothetical protein - NPQTN specific sortase B	Cell wall/membrane biogenesis
lmo2182	hypothetical protein - Heme transporter analogous to IsdDEF, ATP-binding protein	Inorganic ion transport and metabolism; Coenzyme transport and metabolism;
lmo2183	hypothetical protein - Heme transporter IsdDEF, permease component IsdF	Inorganic ion transport and metabolism
lmo2184	hypothetical protein - Heme transporter IsdDEF, lipoprotein IsdE	Inorganic ion transport and metabolism
lmo2185	hypothetical protein - Cell surface protein IsdA, transfers heme from hemoglobin to apo-IsdC	Cell wall/membrane biogenesis
lmo2186	hypothetical protein - NPQTN cell wall anchored protein IsdC	Cell wall/membrane biogenesis
lmo2191	transcriptional regulator Spx	Inorganic ion transport and metabolism
lmo2197	hypothetical protein - FIG00774927: hypothetical protein	Energy production and conversion
lmo2198	tryptophanyl-tRNA synthetase	Translation
lmo2200	hypothetical protein	Transcription
lmo2202	3-oxoacyl-(acyl carrier protein) synthase III	Lipid transport and metabolism
lmo2203	hypothetical protein	Cell motility; Intracellular trafficking and secretion;

lmo2204	hypothetical protein	Not in COGs
lmo2206	hypothetical protein - ClpB protein	Posttranslational modification, protein turnover, chaperones
lmo2210	hypothetical protein - FIG00774091: hypothetical protein	Not in COGs
lmo2212	uroporphyrinogen decarboxylase	Coenzyme transport and metabolism
lmo2213	hypothetical protein	General function prediction only
lmo2230	hypothetical protein - arsenate reductase	Signal transduction mechanisms
lmo2231	hypothetical protein - Cobalt-zinc-cadmium resistance protein	Inorganic ion transport and metabolism
lmo2234	hypothetical protein - Inosose isomerase (EC 5.3.99.-)	Carbohydrate transport and metabolism
lmo2235	hypothetical protein	Energy production and conversion; General function prediction only;
lmo2249	hypothetical protein - Probable low-affinity inorganic phosphate transporter	Inorganic ion transport and metabolism
lmo2260	hypothetical protein - Acetyltransferase, GNAT family, potentially associated with YqeK	Transcription; General function prediction only;
lmo2261	hypothetical protein - hypothetical protein	General function prediction only
lmo2269	hypothetical protein - hypothetical protein	Not in COGs
lmo2271	hypothetical protein - FIG00775160: hypothetical protein	Not in COGs
lmo2275	protein gp28	Not in COGs
lmo2277	hypothetical protein - Lmo2277 protein	Nucleotide transport and metabolism
lmo2279	holin	Not in COGs
lmo2290	protein gp13	Cell motility
lmo2291	major tail shaft protein	Not in COGs
lmo2297	putative scaffolding protein	Cell motility; Signal transduction mechanisms;
lmo2304	bacteriophage A118 gp65 protein	Not in COGs
lmo2305	hypothetical protein - Hypothetical protein, Lmo2305 homolog [Bacteriophage A118]	Not in COGs
lmo2335	hypothetical protein - PTS system, fructose-specific IIA component (EC 2.7.1.69) / PTS system, fructose-specific IIB component (EC 2.7.1.69) / PTS system, fructose-specific IIC component (EC 2.7.1.69)	Carbohydrate transport and metabolism; Signal transduction mechanisms;
lmo2336	fructose-1-phosphate kinase	Carbohydrate transport and metabolism
lmo2337	hypothetical protein - Transcriptional repressor of the fructose operon, DeoR family	Transcription; Carbohydrate transport and metabolism;
lmo2342	hypothetical protein - Ribosomal small subunit pseudouridine synthase A (EC 4.2.1.70)	Translation

lmo2343	hypothetical protein - Coenzyme F420-dependent N5,N10-methylene tetrahydromethanopterin reductase and related flavin-dependent oxidoreductases	Energy production and conversion
lmo2344	hypothetical protein - glutaredoxin family protein	Posttranslational modification, protein turnover, chaperones
lmo2345	hypothetical protein - Bacterial luciferase family protein YtmO, in cluster with L-cystine ABC transporter	Energy production and conversion
lmo2346	hypothetical protein - L-Cystine ABC transporter, ATP-binding protein TcyN	Amino acid transport and metabolism
lmo2347	hypothetical protein	Amino acid transport and metabolism
lmo2348	hypothetical protein - L-Cystine ABC transporter, permease protein TcyL	Amino acid transport and metabolism
lmo2349	hypothetical protein - L-Cystine ABC transporter, periplasmic cystine-binding protein TcyK	Amino acid transport and metabolism; Signal transduction mechanisms;
lmo2350	hypothetical protein - acetyltransferase, GNAT family	Transcription; General function prediction only;
lmo2351	hypothetical protein - FMN reductase (EC 1.5.1.29)	General function prediction only
lmo2352	hypothetical protein - HTH-type transcriptional regulator YtlI, LysR family	Transcription
lmo2356	hypothetical protein - FIG00774030: hypothetical protein	Not in COGs
lmo2357	hypothetical protein - membrane protein	Function unknown
lmo2358	hypothetical protein - Glucosamine-6-phosphate deaminase (EC 3.5.99.6)	Carbohydrate transport and metabolism
lmo2364	hypothetical protein	Not in COGs
lmo2365	hypothetical protein - Listeria RofA-like transcriptional regulator	Transcription; Carbohydrate transport and metabolism;
lmo2371	hypothetical protein - ABC transporter, permease protein	Defense/virulence mechanisms
lmo2375	hypothetical protein - FIG00774641: hypothetical protein	Not in COGs
lmo2386	hypothetical protein - membrane protein	Function unknown
lmo2387	hypothetical protein	Inorganic ion transport and metabolism
lmo2389	hypothetical protein - NADH dehydrogenase (EC 1.6.99.3)	Energy production and conversion
lmo2408	hypothetical protein - DNA-binding protein	Transcription
lmo2409	hypothetical protein - FIG00775866: hypothetical protein	Not in COGs
lmo2410	hypothetical protein - hypothetical protein	Not in COGs
lmo2412	hypothetical protein - Putative iron-sulfur cluster assembly scaffold protein for SUF system, SufE2	Energy production and conversion
lmo2413	hypothetical protein - Cysteine desulfurase (EC 2.8.1.7), SufS subfamily	Amino acid transport and metabolism
lmo2433	hypothetical protein - putative esterase	General function prediction only
lmo2435	hypothetical protein - FIG00774157: hypothetical protein	Function unknown

lmo2436	hypothetical protein	Transcription
lmo2443	hypothetical protein - FIG00774986: hypothetical protein	Not in COGs
lmo2447	hypothetical protein - transcriptional activator	Transcription
lmo2455	phosphopyruvate hydratase	Carbohydrate transport and metabolism
lmo2466	hypothetical protein	Function unknown
lmo2469	hypothetical protein - Amino acid permease	Amino acid transport and metabolism
lmo2484	hypothetical protein - membrane protein	Function unknown
lmo2486	hypothetical protein - FIG00774998: hypothetical protein	Transcription; Signal transduction mechanisms; Function unknown;
lmo2494	hypothetical protein - Phosphate transport system regulatory protein PhoU	Inorganic ion transport and metabolism
lmo2495	phosphate transporter ATP-binding protein	Inorganic ion transport and metabolism
lmo2522	hypothetical protein - Cell wall-binding protein	Cell wall/membrane biogenesis; Function unknown;
lmo2527	hypothetical protein - FIG00774319: hypothetical protein	Function unknown
lmo2573	hypothetical protein - Bifunctional protein: zinc-containing alcohol dehydrogenase quinone oxidoreductase (NADPH:quinone reductase) (EC 1.1.1.-) Similar to arginate lyase	Energy production and conversion; General function prediction only;
lmo2579a	#NV	#NV
lmo2586	hypothetical protein - Formate dehydrogenase related protein	General function prediction only
lmo2587	hypothetical protein - FIG00774290: hypothetical protein	Function unknown
lmo2588	hypothetical protein - lincomycin resistance protein LmrB	Carbohydrate transport and metabolism; Amino acid transport and metabolism; Inorganic ion transport and metabolism; General function prediction only;
lmo2589	hypothetical protein - Transcriptional regulator, TetR family	Transcription
lmo2595	hypothetical protein - lipoprotein	Not in COGs
lmo2609	50S ribosomal protein L36	Translation
lmo2673	hypothetical protein - Universal stress protein family	Signal transduction mechanisms
lmo2675	hypothetical protein - FIG00774830: hypothetical protein	Not in COGs
lmo2676	hypothetical protein	Replication, recombination and repair
lmo2683	hypothetical protein - PTS system, cellobiose-specific IIB component (EC 2.7.1.69)	Carbohydrate transport and metabolism
lmo2684	hypothetical protein - PTS system, cellobiose-specific IIC component (EC 2.7.1.69)	Carbohydrate transport and metabolism
lmo2685	hypothetical protein - PTS system, beta-glucoside-specific IIA component (EC 2.7.1.69) PTS system, cellobiose-specific IIA component (EC 2.7.1.69)	Carbohydrate transport and metabolism

**lmo2707**	**hypothetical protein - FIG00774270: hypothetical protein**	**Not in COGs**
**lmo2711**	**hypothetical protein**	**Not in COGs**
**lmo2721**	**putative 6-phosphogluconolactonase**	**Carbohydrate transport and metabolism**
**lmo2724**	**hypothetical protein - PhnB protein putative DNA binding 3-demethylubiquinone-9 3-methyltransferase domain protein**	**Function unknown**
**lmo2746**	**hypothetical protein**	**Transcription**
**lmo2747**	**seryl-tRNA synthetase**	**Translation**
**lmo2753**	**hypothetical protein - Lin2896 protein**	**Not in COGs**
**lmo2758**	**hypothetical protein - Inosine-5'-monophosphate dehydrogenase (EC 1.1.1.205)**	**Nucleotide transport and metabolism; General function prediction only;**
**lmo2760a**	**#NV**	**#NV**
**lmo2778**	**hypothetical protein - FIG00774119: hypothetical protein**	**Not in COGs**
**lmo2785**	**catalase**	**Inorganic ion transport and metabolism**
**lmo2804**	**hypothetical protein - FIG00774232: hypothetical protein**	**Not in COGs**
**lmo2811**	**tRNA modification GTPase TrmE**	**General function prediction only**
**lmo2826**	**hypothetical protein**	**Carbohydrate transport and metabolism; Amino acid transport and metabolism; Inorganic ion transport and metabolism; General function prediction only;**
**lmo2827**	**hypothetical protein**	**Transcription**
**lmo2828**	**hypothetical protein - FIG00774082: hypothetical protein**	**Not in COGs**
**lmo2829**	**hypothetical protein - Nitroreductase family protein**	**General function prediction only**
**lmo2852**	**hypothetical protein - FIG00774123: hypothetical protein**	**Function unknown**
**lmo2854**	**hypothetical protein - Inner membrane protein translocase component YidC, short form OxaI-like**	**Intracellular trafficking and secretion**
**lmo2855**	**ribonuclease P**	**Translation**
**lmo2856**	**50S ribosomal protein L34**	**Translation**
**lmo2857**	**hypothetical protein - FIG00774274: hypothetical protein**	**Not in COGs**

#NV = No Value
