# Supplementary material for: The secRNome of Listeria monocytogenes Harbors Small Noncoding RNAs That Are Potent Inducers of Beta Interferon
Source: mBio. 2019 Oct 8;10(5):e01223-19. doi: 10.1128/mBio.01223-19 (PMC6786865; doi:10.1128/mBio.01223-19)
Supplement: TABLE S3 [file mBio.01223-19-st003.pdf]

Table S3. Primers used in this study

Primers used for the construction of rli32 overexpression vector

Undrelined sequences are overlaps for the fusion PCR of rli32, promoter and terminator

**Primer**	**Sequence (5`-3`)**	Restriction sites
hly -p_for	GCGCGTCGACGTGACTTTTATGTTGAGGCA	*Sal* I
hly -p_rev	GCTCTCCACAGCTTGCTTTATAGCTTTAT	
rli32_for	TAAAGCAAGCTGTGGAGAGCTTTCATTTTT	
rli32_rev	ACTTTTACAAAAAAAAATAACCGCACCAGG	
hly-T_for	TTATTTTTTTTTGTAAAAGTAATAAAAAATT	
hly-T_rev	GCGCCCCGGGGCTTATATTATATGGATAAA	*Xma* I

Primers used for the generation of Δrli32

**Primer**	**Sequence (5`-3`)**
rli32-1_rev	TCCTATACCAACTATCTGTTCG
rli32-2_for	GTTAAGGGAGATAAACAAGTATATAAAAAATG
rli32-3_rev	ACTTGTTTATCTCCCTTAACCGCATCCCCCT
rli32-4_for	TGTTTCAAACACTCGAGCTGC

Primers used for the generation of *in vitro* transcribed RNA
The T7-RNA polymerase promoter is underlined

**Primer**	**Sequence (5`-3`)**
rli32_for	TAATACGACTCACTATAGGTGTGGAGAGCTTTCATTTTT
rli32_rev	AAAAAAATAACCGCACCAGG
rli48_for	TAATACGACTCACTATAGGAAAGGCATGTTATAATTTAT
rli48_rev	CACTCTTGCAGTAGCGCTGA
rli50_for	TAATACGACTCACTATAGGTTTCTTATGCTATAATAAATTT
rli50_rev	ACATGACTCCCTGAGCAGTAG
rli51_for	TAATACGACTCACTATAGGATATCCCAAAGTTTAAGCCAC
rli51_rev	AAACTAAGTTTAAGCCACCT
rli56_for	TAATACGACTCACTATAGGGAGCACAAAGACGTGTGGAA

rli56_rev	ATAGAAAGAGCCCCTTAAAA
rli60_for	TAATACGACTCACTATAGGATTTTTTTCAAAAATGTGCGAC
rli60_rev	TTTTCGATGCTTGAAAACTCG
rli62_for	TAATACGACTCACTATAGGTGTGATTGGTTATGATAATT
rli62_rev	ACGGAGCATAATAAAATAAGG
rli90_for	TAATACGACTCACTATAGGAGTAAAAAGAGAGACAATTG
rli90_rev	TTATTGTATAGAAAAGCACG
rli98_for	TAATACGACTCACTATAGGGAAGGATGTTTTAGAGGAAA
rli98_rev	GTGAAAGGCATGTTATAATT
rli99_for	TAATACGACTCACTATAGGTTCGGATTTAAGGTATAATT
rli99_rev	ATTTAGAGGAATTAGAGCGT
rli100_for	TAATACGACTCACTATAGGAAAAATCAGAATTCATAGTAC
rli100_rev	GCTGGTGGCTAATAAGGGACT
rli105_for	TAATACGACTCACTATAGGCAAGACTGTTAGAATAGGGA
rli105_rev	TTTCAAGACTATTAAACTAGC
rli108_for	TAATACGACTCACTATAGGGCTATCATTAGTAGTATTTT
rli108_rev	TTAAAAGTAAGGGAGCGCTG
rli111_for	TAATACGACTCACTATAGGGCTCATTTCTAAGGATGACT
rli111_rev	TGGGCTGTATGAAAAAACATC
rliG_for	TAATACGACTCACTATAGGGATGACGACACTTCTGTTCA
rliG_rev	CAACAGATGGAAAGGGCTAT
LhrA_for	TAATACGACTCACTATAGGGAACAATAGTAAAATAAGTT
LhrA_rev	CATTTCCAGCGTTGCCATCA
rnpB_for	TAATACGACTCACTATAGGTTCCGGCAGAAATGCTCGGA
rnpB_rev	AAAAAGCTGTTTTAAGAGGG
SRP_for	TAATACGACTCACTATAGGTTTATTTGGCATTTAATTAT

SRP_rev	AGCAAAATAGTCTTTTATAT
ssrA_for	TAATACGACTCACTATAGGTTTTTCCTGTATAATAACTA
ssrA_rev	GTTTTCTTGACTCCATCGTT
ssrS_for	TAATACGACTCACTATAGGAGAAAAGAAACCCTAATGTA
ssrS_rev	CAAAAAAGAAACCCCAATCG
lmot67_for	TAATACGACTCACTATAGGAGTATCACCTTGACATGGT
lmot67_rev	AAAGGGATGCTTTTTTGATAAATG
lmot11_for	TAATACGACTCACTATAGGGGACAGACTCGAAATCTGTT
lmot11_rev	TCTACGGAGAAACGGGGATT
RNA motif 1_for	TAATACGACTCACTATAGGGCTTTCATTTTTCCCAAGAGAAAGC
RNA motif 1_rev	GCTTTCTCTTGGGAAAAATGAAAGCCCTATAGTGAGTCGTATTA
RNA motif 2_for	TAATACGACTCACTATAGGAATAATGTTAGAGCAATAA
RNA motif 2_rev	AATAATATTAAAGCGAGCCA
RNA motif 3_for	TAATACGACTCACTATAGGCCTAATACTCCCTTAACCGCATCCCCCTGG TGCGGTTATTTTTTT
RNA motif 3_rev	AAAAAAATAACCGCACCAGGGGGATGCGGTTAAGGGAGTATTAGGCCT ATAGTGAGTCGTATTA

Primers used for in vitro transcriptiom of motif 2 modifications

Primer	Sequence (5`-3`)
rli32 RNA-Motif-2-for	TAATACGACTCACTATAGGGATACTGTTAGAGCAATAATT
rli32 RNA-Motif-2-rev-1	AAGAAGATTAAAGCGAGCCAAG
rli32 RNA-Motif-2-rev-2	AAGAAGATTATAGCGAGCCAAG

Primers used for the construction of rli32 expression vector under the control of its native promoter

Primer	Sequence (5`-3`)	Restriction sites
Undrelined sequences are overlaps for the fusion PCR of rli32, promoter and terminator		
rli32_ for	GCGCGTCGACAAAAACATTGGCTTG	*Sal* I
rli32_rev	GCGCGGATCCAAACATGACATTTTCGTAAC	*BamH* I

hly-T_for	TTATTTTTTTTTGTAAAAGTAATAAAAAATT	
hly-T_rev	GCGCCCCGGGGCTTATATTATATGGATAAA	*Xma* I
